# An integrated SAGA and TFIID PIC assembly pathway selective for poised and induced promoters

**DOI:** 10.1101/gad.350026.122

**Published:** 2022-09-01

**Authors:** Chitvan Mittal, Olivia Lang, William K.M. Lai, B. Franklin Pugh

**Affiliations:** 1 Department of Biochemistry and Molecular Biology, Pennsylvania State University, University Park, Pennsylvania 16801, USA;; 2 Department of Molecular Biology and Genetics, Cornell University, Ithaca, New York 14850, USA

**Keywords:** *Saccharomyces*, genomics, ChIP-seq, transcription preinitiation complex, gene regulation, SAGA

## Abstract

Genome-wide, little is understood about how proteins organize at inducible promoters before and after induction and to what extent inducible and constitutive architectures depend on cofactors. We report that sequence-specific transcription factors and their tethered cofactors (e.g., SAGA [Spt–Ada–Gcn5–acetyltransferase], Mediator, TUP, NuA4, SWI/SNF, and RPD3-L) are generally bound to promoters prior to induction (“poised”), rather than recruited upon induction, whereas induction recruits the preinitiation complex (PIC) to DNA. Through depletion and/or deletion experiments, we show that SAGA does not function at constitutive promoters, although a SAGA-independent Gcn5 acetylates +1 nucleosomes there. When inducible promoters are poised, SAGA catalyzes +1 nucleosome acetylation but not PIC assembly. When induced, SAGA catalyzes acetylation, deubiquitylation, and PIC assembly. Surprisingly, SAGA mediates induction by creating a PIC that allows TFIID (transcription factor II-D) to stably associate, rather than creating a completely TFIID-independent PIC, as generally thought. These findings suggest that inducible systems, where present, are integrated with constitutive systems.

## Abstract (precis)

In this study, Mittal et al. report that sequence-specific transcription factors and their tethered cofactors (e.g., SAGA, Mediator, TUP, NuA4, SWI/SNF, and RPD3-L) are generally bound to promoters prior to induction (“poised”), rather than recruited upon induction, whereas induction recruits the preinitiation complex (PIC) to DNA. Their findings suggest that inducible systems, where present, evolved on top of constitutive systems.

Budding yeast have evolved constitutive promoter regulatory mechanisms that maintain housekeeping levels of gene expression (typically low levels) in essentially any condition that supports growth. They have also evolved inducible systems at specific genes that allow a wide range of expression when faced with distinct environmental conditions (Struhl 1986; Rossi et al. 2021). We recently described the individual protein–DNA architectures of these systems at very high positional resolution across the *Saccharomyces* (budding yeast) genome (Rossi et al. 2021). This prior work did not distinguish induced versus uninduced architectures or address the functional importance of these architectures and their relationships to constitutive genes. Here we describe multiple types of architectures and how inducible systems are integrated with constitutive systems. Since the major architectural components are conserved across eukaryotes, these findings offer a model for gene regulatory mechanisms across eukaryotes.

Previously, we defined 5378 yeast promoters with measurable RNA polymerase (Pol) II preinitiation complexes (PIC) in rich media (Rossi et al. 2021). A PIC consists of Pol II and general transcription factors (GTFs) such as TBP (Spt15), TFIIB (Sua7), and others (Cramer 2019). About 20% of promoters are classified as inducible (Rossi et al. 2021). In their upstream promoter region, inducible promoters possess upstream activating sequences (UASs) for sequence-specific transcription factors (ssTFs) that are effectors of environmental sensing. ssTFs recruit cofactors such as histone-modifying complexes (SAGA [Spt–Ada–Gcn5–acetyltransferase], RPD3-L, and NuA4) and nucleosome remodeling complexes (SWI/SNF), presumably to regulate local chromatin accessibility at their nucleosome-depleted promoter regions (NDRs). They might do so to varying extents in an induced versus an uninduced state. Thus, induced promoters are expected to have highly active ssTFs, cofactors, and PICs that produce high transcriptional output. Uninduced promoters may be more ambiguously defined as having none, some, or all of components of induced promoters but lacking the full transcriptional output of an induced gene.

Constitutive promoters (∼80% of all promoters) lack this inducible architecture (Rossi et al. 2021). They generally do not have ssTF binding sites, except where certain ssTFs (such as Reb1, Abf1, and Rap1) act as insulators against upstream events. Insulators also help position flanking nucleosomes into a common framework of chromatin organization (Hartley and Madhani 2009; Krietenstein et al. 2016). This results in a small (<150-bp) constitutively nucleosome-free region (NFR) that provides constitutive access to GTFs for low levels of PIC assembly. By this definition, NFRs are structurally distinct from NDRs (constitutively nucleosome-free vs. ssTF-mediated depletion).

The concept of constitutive and inducible promoters dovetails with the notion of two distinct PIC assembly pathways either through TFIID (transcription tactor II-D) or through SAGA (Moqtaderi et al. 1996; Walker et al. 1996; Kuras et al. 2000; Li et al. 2000; Huisinga and Pugh 2004, 2007; Tirosh and Barkai 2008; Donczew et al. 2020). While the details remain unclear, both mechanisms converge on the recruitment of TBP to promoters, along with the remaining GTFs (Timmers 2021). Where present, at least some ssTFs engage SAGA and other cofactors to augment TBP delivery to promoters (Dudley et al. 1999; Bryant and Ptashne 2003; Qiu et al. 2005; Sermwittayawong and Tan 2006; Papai et al. 2020; Rossi et al. 2021). TBP-associated factors (TAFs) also recruit TBP to promoters through the formation of the TFIID complex (Burley and Roeder 1996). TAF dependency (TAF_dep_) appears to dominate most but not all promoters (Huisinga and Pugh 2004; Warfield et al. 2017). Although not firmly established, TFIID might gain promoter specificity by recognizing some aspect of the NFR/+1 nucleosome architecture (Matangkasombut et al. 2000; Vermeulen et al. 2007; Bhuiyan and Timmers 2019; Chen and Xu 2022). Importantly, many induced promoters recruit substantially more GTFs than constitutive promoters and have unusually high ratios of GTFs to TAFs (Kuras et al. 2000; Li et al. 2000; Rossi et al. 2021). High GTF:TAF ratios have been hallmarks of induced, or so-called TAF-independent (TAF_ind_), SAGA-dominated promoters (Kuras et al. 2000; Li et al. 2000, 2002; Huisinga and Pugh 2004). An alternative view that SAGA functions as a general cofactor at essentially all genes, rather than being gene-specific, has received considerable attention (Bonnet et al. 2014; Baptista et al. 2017; Taatjes 2017; Warfield et al. 2017) but has not been fully confirmed (Donczew et al. 2020; Mittal et al. 2021). Here we comprehensively examine the gene specificity of SAGA's multiple activities in the context of distinct promoter classes.

While constitutive promoters are simple and largely homogenous in factor organization and regulation, inducible promoters are quite diverse (Rossi et al. 2021). In the present study, we therefore created four classes of inducible promoters that reflect this diversity. They include induced, poised, and condition-specific (heat shock) promoters, and the uniquely regulated ribosomal protein (RP) promoters. We examined the genome-wide positional organization of >400 different proteins at each gene class to identify class-specific architectures. We then focused deeply on defining the multiple activities of SAGA in these systems. SAGA activities include activator interactions (Brown et al. 2001; Govind et al. 2005; Lin et al. 2012), augmented TBP delivery (Bhaumik and Green 2002; Papai et al. 2020), H3K9 acetylation (Brownell et al. 1996; Grant et al. 1997), and H2B deubiquitylation (Henry et al. 2003; Daniel et al. 2004).

We found that SAGA's TBP delivery activity is more restricted than even the earliest studies had suggested (Huisinga and Pugh 2004), being largely limited to induced promoters, which represent only ∼8% of all promoters in rich media. However, we found that SAGA and other cofactors nevertheless engage with ssTFs at the UAS of inducible promoters that are not induced (i.e., have low levels of PIC assembly). This class, termed “poised,” represents ∼15% of all promoters. SAGA acetylates their +1 nucleosomes but has more limited ability to augment TBP delivery when these promoters are poised. We propose a new mechanism at induced promoters where SAGA and TFIID are interdependent in building a single highly active PIC. SAGA-built PICs are recognized by TAFs to form a TFIID PIC. This differs substantially from current but untested models of distinct TAF_ind_ and TAF_dep_ PICs. In contrast, SAGA is neither present nor appreciably active at constitutive promoters. At constitutive promoters, TAFs are largely left to their own devices to recruit low levels of TBP, and this is assisted by Gcn5-dependent (not SAGA) acetylation of the +1 nucleosome.

## Results

### Classes of inducible promoters

**Figure 1.**
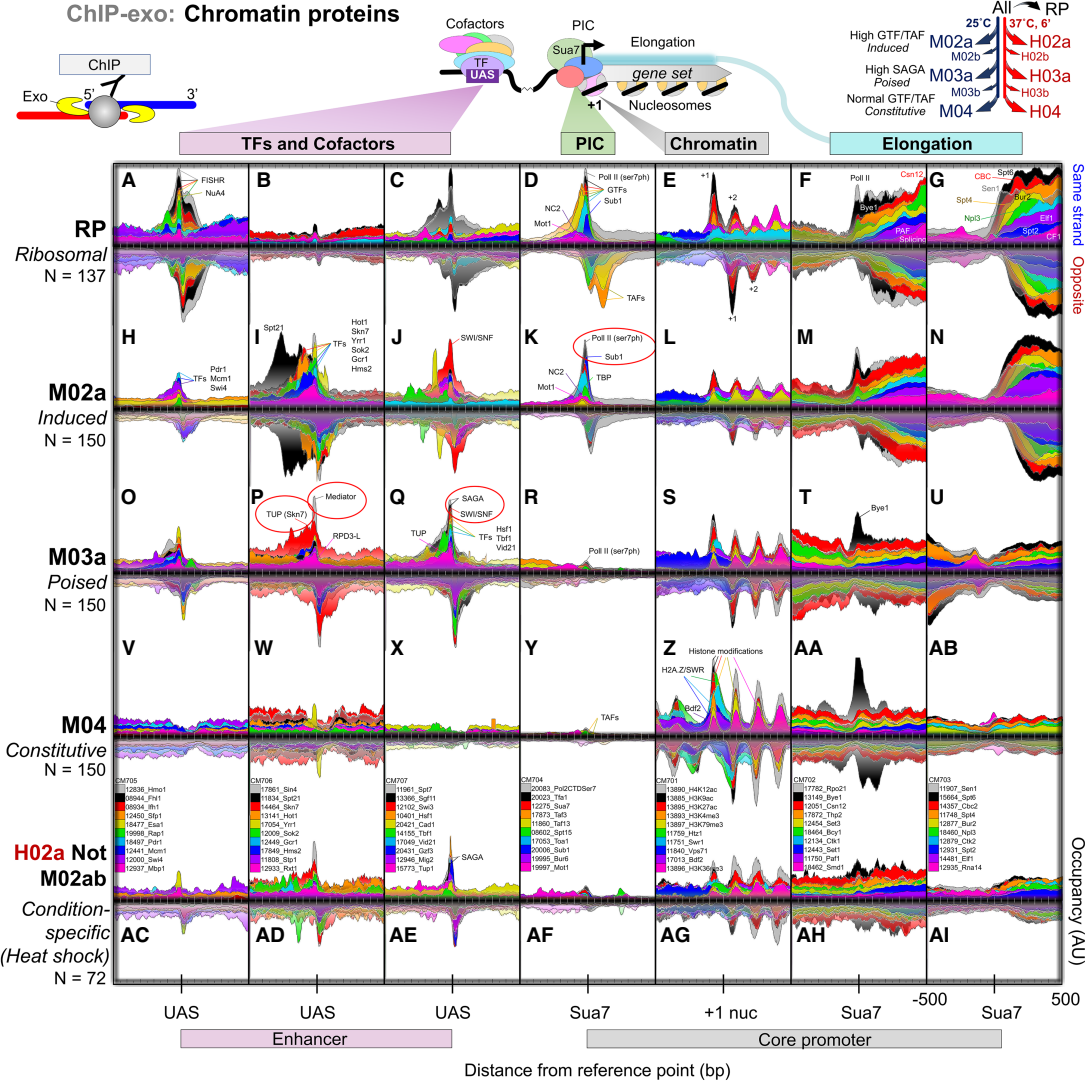
Protein architectures of constitutive and inducible (RP, induced, poised, and condition-specific) promoters. Composite plots of chromatin immunoprecipitation exonuclease (ChIP-exo) tag 5′ ends (exonuclease stop sites as illustrated) for 70 representative promoter/gene-associated proteins (out of 400) are shown distributed around the indicated *X*-axis reference point (UAS, Sua7, or +1 nuc) within each promoter class (rows of panels). Each target protein in a vertical column is defined by one color whose legend is at the *bottom*. Selected complexes that are represented by a protein are pointed out in particular panels. See the Materials and Methods for details. *X*-axis intervals are orientated such that transcription proceeds to the *right*. UAS corresponds to a ssTF binding site or the equivalent distance upstream (150 bp) of a transcription start site (TSS) when no UAS was present. Sua7 (TFIIB) corresponds to the Sua7 ChExMix peak closest to an annotated TSS and is essentially the PIC location. +1 nuc is the downstream nucleosome midpoint closest to a TSS. Alignment to these reference features provided high positional resolution. Opposite-strand data are inverted. “*N*” denotes class membership count. The *Y*-axis occupancy scale is the same and thus comparable for a particular target across the different promoter classes (analysis ID: CM701–CM707).

To provide granularity on inducible versus constitutive regulatory mechanisms on a genomic scale, we established the following five promoter classes representing broad but distinct archetypes of regulation: (1) RP, (2) induced, (3) poised, (4) constitutive, and (5) condition-specific (induced by heat shock). Since the constitutive class is quite distinct from the other four classes, we collectively refer to the latter set as “inducible.” These labels are metaphors for very specific selection criteria used in this study. They are not exclusive of other criteria that could use the same labels. We aimed to test the hypotheses that (1) induced promoters can be characterized simply by relatively high GTF:TAF ratios (i.e., without using ssTFs, cofactors, or transcription rates as criteria), (2) poised promoters can be characterized simply by high SAGA occupancy after removing RP and induced promoters, and (3) constitutive promoters can be characterized as having “normal” GTF:TAF ratios after removing those defined as inducible. This then provides a basis for defining precise protein/DNA architectures and interrelated mechanisms of PIC assembly at inducible versus constitutive promoter classes. Details of membership criteria for the five classes and its justification are provided in the Materials and Methods section. Class membership was capped at *N* = 150 but imputed for all 5378 PIC-containing promoters using multiple criteria (Supplemental Table S1). Briefly, RP promoters were selected first, followed by induced promoters, then poised promoters, and finally constitutive promoters (Fig. 1, top right diagram). The RP class is uniquely coregulated by “FISHR” (Fhl1, Ifh1, Sfp1, Hmo1, and Rap1) and is highly active at 25°C but repressed upon heat shock (Reja et al. 2015). It has Sua7:Taf2 (GTF:TAF) occupancy ratios equivalent to those of constitutive promoters (Reja et al. 2015; Rossi et al. 2021) but also has properties of induced promoters. The “induced” class was defined as having high TBP occupancy and the highest Sua7:Taf2 occupancy ratios at 25°C (algorithm ID: M02a). The presence of cofactors was not a criterion. The “poised” class was defined as having the highest SAGA occupancy at 25°C after removing RP and induced promoters (algorithm ID: M03a). The “constitutive” class was defined as being closest to the genome-wide mode for Sua7:Taf2 ratios after removing the prior classes (algorithm ID: M04). The “condition-specific” promoter class was defined as being heat shock-induced but not induced at 25°C (algorithm ID: H02aNotM02ab). About half of these overlapped with the poised class (Supplemental Table S1). They represent poised promoters that demonstrably can be induced.

### Protein architectures of constitutive and inducible (RP, induced, and poised) promoters

To assess the promoter and gene body architecture of proteins at the five promoter classes, we examined >1000 chromatin immunoprecipitation exonuclease (ChIP-exo) data sets representing ∼400 different proteins in ∼40 genome-wide meta-assemblages (Supplemental Table S2; Rossi et al. 2021). The ChIP-exo assay concentrates protein–DNA interaction signals separately on each DNA strand in a very narrow genomic window where cross-linking occurs (Fig. 1, top left diagram), ensuring that background contributions are minimal (Rhee and Pugh 2011). Figure 1, A–AI, displays the strand-separated ChIP-exo patterning of a diverse representative subset of these proteins (*N* = 70, 10 per column) organized across the five promoter classes (rows) and aligned by their most relevant promoter-organizing center: UAS, Sua7, or +1 nucleosome. Sua7 (TFIIB) peak locations were used to provide high-accuracy PIC locations, since yeast transcription start sites (TSSs) are more variable due to Pol II template scanning prior to productive initiation (Kuehner and Brow 2006; Qiu et al. 2020). In all cases, ChIP-exo patterns were confirmed with replicates and/or different components of the same complex. Below, we describe proteins that were enriched and highly positioned within these promoter classes, with some being class-specific.

Constitutive promoters essentially lacked ssTFs and cofactors despite their absence not being criteria for their selection (Fig. 1V–X). As expected (Reja et al. 2015), RP-specific regulators (“FISHR”) were restricted to RP promoters and possessed a ChIP-exo pattern that reflects “FISH” being positionally anchored at Fhl1/Hmo1 DNA recognition sequences but also connected to neighboring Rap1 (Fig. 1A). Histone acetyltransferase complexes NuA4 (Esa1) and SAGA (Spt7 and Sgf11) were enriched and precisely positioned with ssTF Rap1 but not with Fhl1/Hmo1 (Fig. 1A,C; Supplemental Table S3). Both NuA4 and SAGA contain Tra1, which interacts with ssTFs (Allard et al. 1999; Brown et al. 2001; Reeves and Hahn 2005; Lin et al. 2012). Further downstream (∼200 bp), the expected products of NuA4 and SAGA (Bdf1/2 binding and H3K9ac, respectively) were highly enriched and positioned at RP +1 nucleosomes (Fig. 1E). Conspicuously depleted at these highly active RP promoters were the subunits of Mediator, SWI/SNF, RPD3-L, and TUP cofactors (Fig. 1B,C vs. P,Q). We demonstrated this further by examining all parts of Mediator (tail, middle, head, and kinase modules) (Supplemental Fig. S1A), and this is further confirmed by earlier studies (Fan et al. 2006). Therefore, Rap1 stably engages SAGA and NuA4 but not Mediator, SWI/SNF, RPD3-L, and TUP cofactors at the RP class.

To address whether a transient Mediator interaction could be detected, we turned to ChIP-exo data from a TFIIH *kin28as* mutant that inhibits promoter escape and results in increased PIC and Mediator occupancy at core promoters (Jeronimo and Robert 2014). Compared with a wild-type *KIN28* strain, Mediator (Med15 and Med7) was slightly enriched at RP core promoters in the *kin28as* mutant (Supplemental Fig. S1B). However, this enrichment was very low compared with the high Mediator enrichment at the equivalently expressed induced promoter class. Mediator at RP promoters was on par with the very low amount detectable at constitutive core promoters. At all promoter classes, Pol II (Rpb3) enrichment in the *kin28as* mutant relative to WT was high at the core promoter and also 100–200 bp downstream from the PIC. Both regions are where Pol II stalls during stress (Rodríguez-Molina et al. 2016; Badjatia et al. 2021), with the latter also being where Pol II picks up many elongation factors. In the *kin28as* mutant, Pol II was strongly enriched at the core promoter for the induced class compared with all other classes, much like Mediator. We interpret this as promoter escape being more Kin28-dependent at induced promoters, as previously reported (Wong et al. 2014). These results confirm that Pol II rapidly moves out from the core promoter and through the stall region of all promoter classes in a Kin28-enhanced manner. With Pol II escape being more dependent on Kin28 at induced core promoters, Mediator release is therefore also more Kin28-dependent at induced promoters, in accord with prior studies (Jeronimo and Robert 2014; Wong et al. 2014). This analysis is consistent with Mediator acting at all core promoters but with a very short dwell time (and thus detectability) compared with induced promoters.

**Figure 2.**
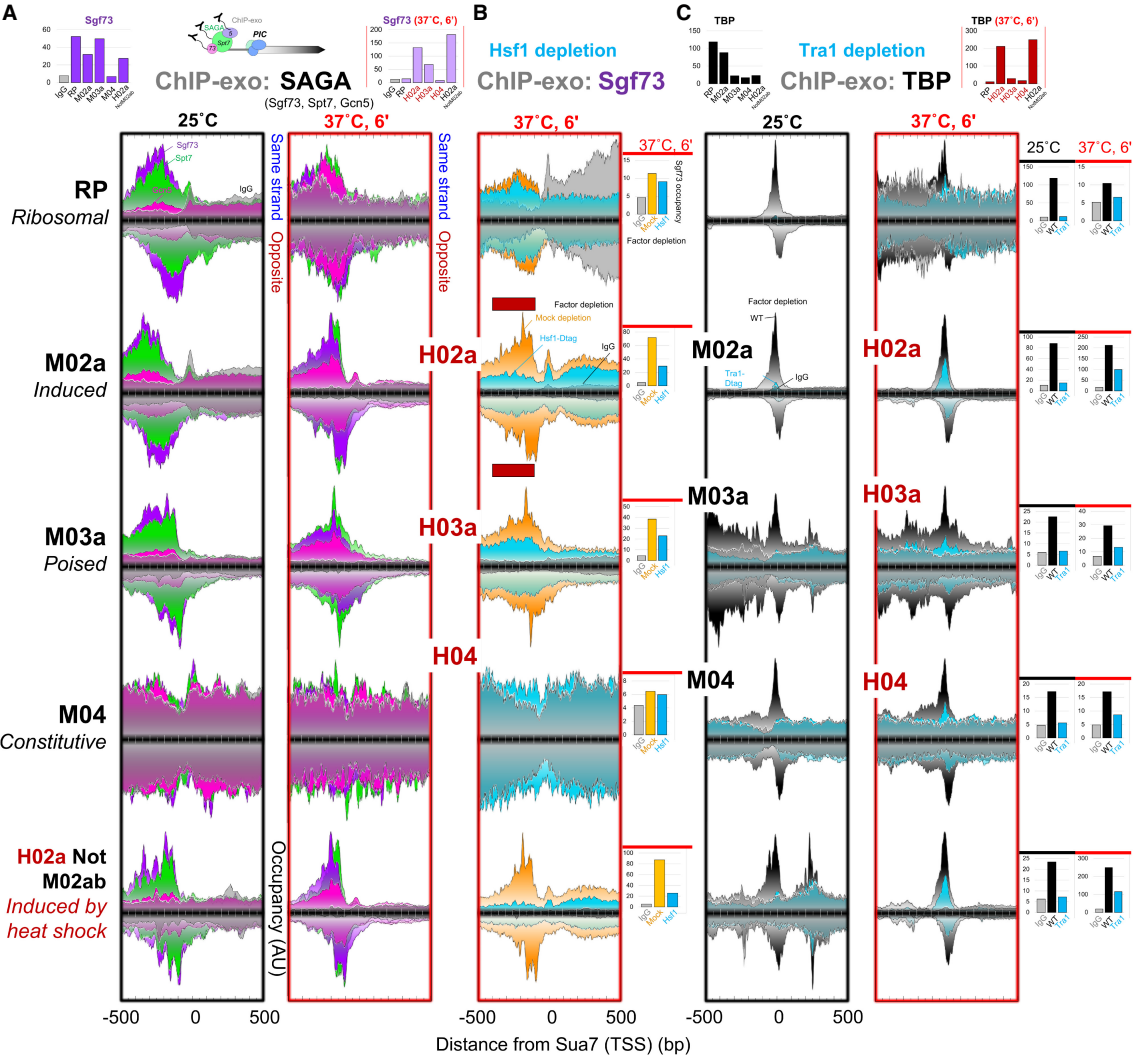
SAGA binding at UASs depends on Hsf1, and TBP binding depends on Tra1. Composite plots of ChIP-exo tag 5′ ends of the indicated proteins (SAGA or TBP) are distributed around the Sua7 reference point within each promoter class at the indicated cellular temperature (25°C; 6 min at 37°C). Each fill color represents a target or mutant that is pointed out in a panel. See Figure 1 and the Materials and Methods for plotting details. The *Y*-axis occupancy scale is linear and the same for a particular target (or control) within a promoter class for both mutant and WT and is quantified in the bar graphs at the *right* of the relevant panel set. Quantified regions in the bar graphs are demarcated by the red bars in traces in *B*. The *Y*-axis occupancy is scaled separately among promoter classes to maximize visual distinction and thus (unlike Fig. 1) is not directly comparable. Their comparable WT values are provided in the bar graphs at the *top*. Consequently, RP (6 min at 37°C) and M04 plots show essentially background SAGA occupancy that is magnified on the *Y*-axis (see IgG control). (*A*) Positional organization of SAGA subunits upstream of the PIC. Bar graph quantification is relative to the reference point (Sua7) from −400 to −100 on the top (TSS) strand and from −300 to 0 on the bottom strands (analysis ID: CM04 and CM05). (*B*) SAGA (Sgf73) occupancy is shown upon rapid depletion of Hsf1 (cyan fill) or a mock depletion (orange fill) and compared with an IgG-negative control (gray fill) under acute heat shock conditions (6 min at 37°C). (*Right*) Bar graph quantifications are relative to the reference point (Sua7) from −400 to −100 on the top (TSS) and bottom strands (analysis ID: CM18). (*C*) TBP (PIC) occupancy is shown upon rapid depletion of Tra1 (via its Dtag). (*Right*) Bar graph quantifications are relative to the reference point (Sua7) from −70 to +30 on the top (TSS) strand plus from −40 to +60 on the opposite strand (analysis ID: CM47 and CM48).

Compared with RP promoters, the induced promoter class had different ssTFs (e.g., Hot1, Yrr1, Sok2, and Hms2) and cofactors (Mediator, SWI/SNF, RPD3-L, and TUP) (Figs. 1H–J, 2A; Supplemental Table S3). The induced class was also enriched with NuA4 and SAGA but to a lesser extent than RP and the poised class. The poised class had ssTFs (e.g., Hsf1, Tbf1, and Vid21) that largely differed from those at RP and the induced class (Fig. 1O–Q). Remarkably, poised promoters were fully stocked with all the major cofactors (SAGA, NuA4, Mediator, SWI/SNF, TUP, and RPD3-L), as were condition-specific promoters (algorithm ID: H02aNotM02ab in Fig. 1AC–AE). However, both the poised and condition-specific classes had very low PIC assembly that was on par with constitutive promoters (Fig. 1R,AF vs. Y). This relationship remained true when only tandem genes were considered, thereby ruling out effects primarily from divergent promoters. Thus, high occupancy of SAGA, Mediator, and/or other cofactors does not necessarily correspond to high PIC occupancy.

Previously, we proposed that cofactor ChIP-exo signals that were concentrated within a few base pairs of the UAS represented cofactors cross-linking through the ssTF to which it is tethered (Reja et al. 2015; Rossi et al. 2021). This was observed for all inducible promoter classes (Fig. 1A–C,H–J,O–Q). Additionally, broader cross-linking ∼200 bp on either side of the UAS reflected the diffuse proximity of cofactors to the ssTF (Rossi et al. 2021). As this may reflect some conformational or coalesced state of cofactors, we examined whether induced and poised promoters differed in this regard. We measured the average ChIP-exo signal for Mediator subunits within 30 bp of the UAS relative to its broader flanks to generate a “UAS focal ratio” (Supplemental Fig. S1A). This ratio was substantially higher at poised (and uninduced condition-specific promoters) compared with induced promoter classes. Thus, while Mediator and other cofactors are tethered to ssTFs at the UAS (Bhoite et al. 2001; Fan et al. 2006; Kim and Gross 2013; Rossi et al. 2021), Mediator is more broadly distributed around the UAS when it is activating transcription than when it is present in a poised state. This is consistent with other studies (Petrenko et al. 2016) and may reflect broader interactions of the ssTF/Mediator complex with other cofactors and the induced PIC.

Since both positive and negative cofactors were abundant at induced and poised promoter classes, each might be toggled on or off, thereby creating either an induced or a poised state that is reflected in part by (1) the types of ssTFs present, (2) the conformational state of the coalesced cofactors, (3) the level of PIC assembly, and (4) the amount of Pol II transcription. Such toggling has been demonstrated for Tup1 (Proft and Struhl 2002; Wong and Struhl 2011). This explains why TF/cofactor occupancy and gene expression dependency are often unlinked (Lenstra and Holstege 2012). Together, these results suggest that regardless of their state of induction, many ssTFs bind their cognate UASs at inducible promoters and tether cofactors. However, RP promoters bring in certain cofactors (e.g., SAGA and NuA4), whereas induced and poised promoters bring in all major cofactors (SAGA, NuA4, Mediator, SWI/SNF, TUP, and RPD3-L). ssTFs may be tied more to specific cofactors than to others (e.g., Skn7 to Mediator or Sok2 to TUP) (Rossi et al. 2021). Other condition-specific ssTFs such as Gal4 may be engaged primarily with repressive cofactors such as Gal80 (in glucose media) that preclude interactions with the major cofactors and thus are repressed and not poised (Lue et al. 1987; Ma and Ptashne 1987).

RP, induced, and poised promoter classes had additional characteristics, such as the binding of lesser-studied proteins at the PIC site. For example, induced PICs had particularly high Sub1 occupancy (Fig. 1K). Sub1 has been implicated in binding to melted promoter DNA (Sikorski et al. 2011) and suppressing mutations in TFIIB. Sub1 might therefore have a special regulatory role at induced promoters. Relative to PIC levels, we found poised and constitutive classes to be strongly enriched with Bye1 at the PIC (or +1 nucleosome) location despite PICs being largely absent there (Fig. 1T,AA vs. M). The organization of Bye1 on genes is unique in being robustly present at the start and end of genes but not generally present during elongation except at RP genes (Rossi et al. 2021). Bye1 overexpression suppresses mutations in the Ess1 elongation factor and negatively regulates early elongation (Wu et al. 2003; Harlen and Churchman 2017). We also observed relatively low levels of the elongation regulator Spt2 (not Spt20) at RP promoters compared with the induced class, which adds to the distinctiveness of RP regulation compared with other induced genes (Fig. 1G,N).

The constitutive class, despite lacking ssTFs and stably bound cofactors (e.g., SAGA, TUP, SWI/SNF, etc.), was enriched with well-positioned genic nucleosomes that have an abundance of transcription-linked histone marks (H3K9ac, H4K12ac, H3K27ac, H3K4me3, and H3K36me3), H2A.Z/SWR, and Bdf1/2 than would be expected of their low levels of PIC occupancy and transcription (Fig. 1Z). Thus, many transcription-linked chromatin marks are long-lived.

### Hsf1 tethers SAGA to poised and induced promoters

To define the functional interplay among factors within inducible and constitutive architectures, we focused on the well-studied but unresolved relationship between SAGA and TFIID during PIC assembly. We examined TFIID and the four activities of SAGA on a genomic scale using deletion and/or depletion mutants and then measured protein–DNA architectures at the five promoter classes, along with impacts on nascent transcription. Strain validation, system controls, and data quality assessments are described in the Supplemental Material, including Supplemental Figures S2 and S3A, and Supplemental Table S4. In addition to examining inducible and constitutive promoter states under normal growth conditions (25°C, rich media, and M-series of promoter classes), we examined responses to acute heat shock (6 min at 37°C and H-series algorithm of promoter classes) to assess the role of SAGA modules and TFIID in rapid genome-wide reprogramming. To this end, membership in the H-series of promoter classes (algorithm IDs: H02a, H03a, and H04) paralleled the M-series criteria except being defined under acute heat shock. All depletions were conducted for 30 min at 25°C before applying heat shock (6 min at 37°C) or a mock equivalent (6 min at 25°C).

Since we examined genome reprogramming by heat shock, we selected Hsf1 as a representative ssTF for functional contribution. As expected, Hsf1 bound the UAS region of heat shock-induced promoters (H02a and H02aNotM02ab) before and after heat shock and was not enriched at other promoter classes (Supplemental Fig. S4). SAGA was precisely centered on Hsf1-bound sites and UASs at other inducible promoter classes such as RP.

Both Hsf1 and SAGA occupancy increased at induced promoters upon heat shock. We tested whether Hsf1 recruits SAGA by rapidly depleting Hsf1 at 25°C and then measuring SAGA (Sgf73) occupancy after 6 min of heat shock. SAGA became markedly depleted only at promoter classes that were normally enriched with Hsf1 (Fig. 2B). In contrast, when SAGA and NuA4 were depleted via Tra1, there was little or no loss of Hsf1 occupancy relative to mock (Supplemental Fig. S4). Thus, SAGA occupancy depends on promoter-bound Hsf1, but Hsf1 occupancy does not depend on SAGA and NuA4. NuA4 plays a central role in TFIID-directed PIC assembly (Fishburn et al. 2005; Knutson and Hahn 2011; Uprety et al. 2015), possibly by providing histone acetyl docking sites for TFIID via Bdf1 (Matangkasombut et al. 2000). With TFIID and NuA4 playing an essential role at most promoters, Tra1 in NuA4 is expected to be central to all classes of promoters. Indeed, depletion of Tra1 resulted in severe loss of TBP at all promoter classes under normal and heat shock conditions (Fig. 2C). Thus, Tra1 is important to both constitutive and inducible PIC assembly pathways.

### SAGA primarily drives induced PIC assembly

**Figure 3.**
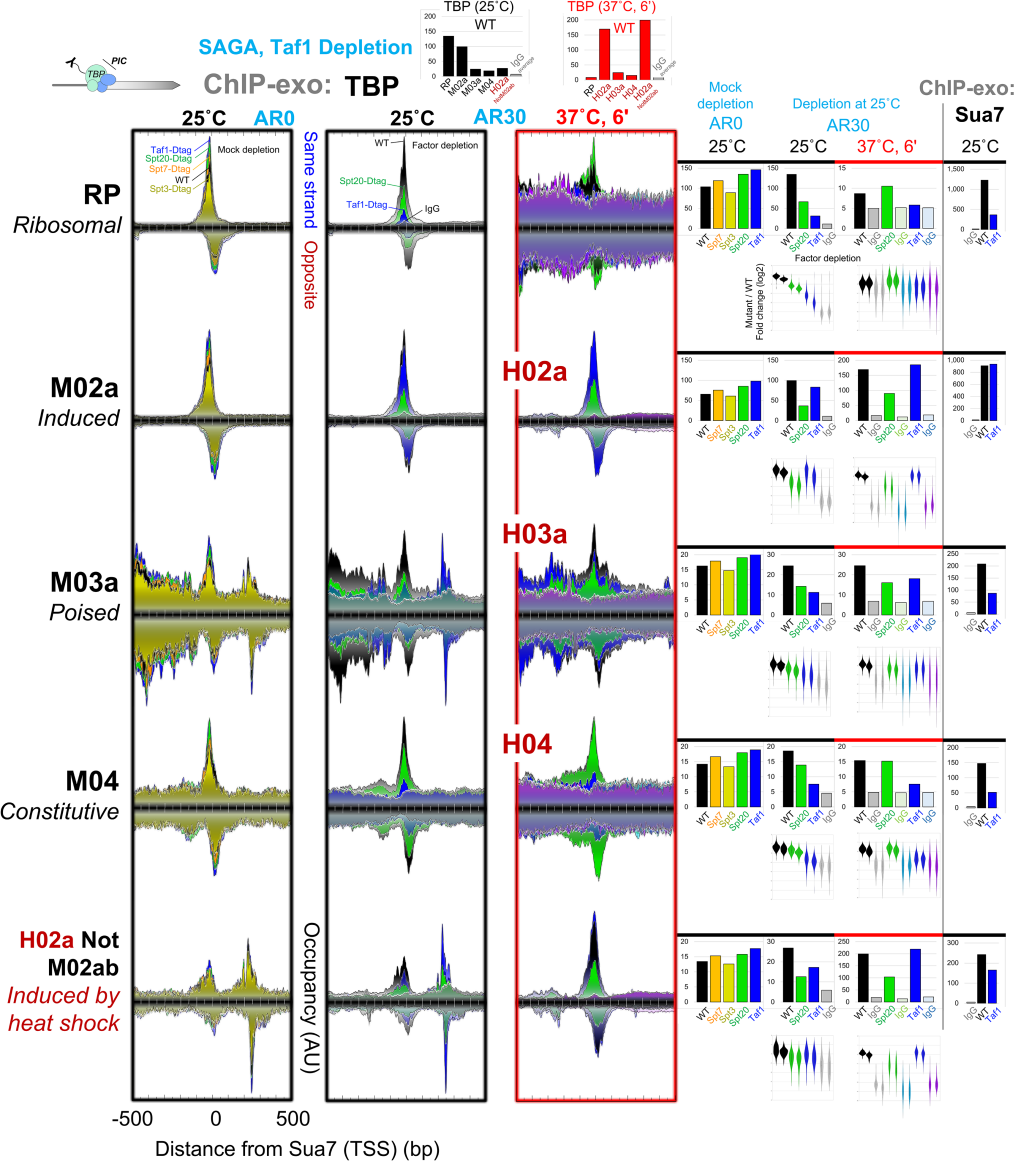
SAGA primarily drives induced PIC assembly. Composite plots are shown of ChIP-exo tag 5′ ends of TBP upon SAGA (Spt20) or TFIID (Taf1) depletion, distributed around the Sua7 reference point within each promoter class at the indicated cellular temperature (25°C; 6 min at 37°C). See Figure 1 and the Materials and Methods for plotting details. Also shown are violin plots of log_2_ fold changes of TBP occupancy of mutant over WT for individual promoters within each class. Two biological replicates are shown for each depletion mutant and independent WT and IgG controls. Bar graph quantification of Sua7 ChIP-exo conducted in parallel is shown at the far *right* (analysis ID: CM30, CM01, and CM02).

We next examined to what extent PIC assembly was dependent on SAGA (Spt20 or Spt7) and TFIID (Taf1) at the five promoter classes. As expected, the induced and constitutive classes had the highest and lowest levels of TBP occupancy, respectively, in accord with their expression level and the transient nature of constitutive PICs (Fig. 3, top bar graphs; Nguyen et al. 2021). Either depletion or deletion of SAGA at 25°C resulted in substantial loss of PICs (TBP/Spt15) at induced promoters, whether induced at 25°C or at 37°C (Fig. 3; Supplemental Fig. S3A,B). Little or no loss of PICs was observed at constitutive promoters. The same conclusions were drawn when all promoters were examined (Supplemental Fig. S5A). This fits with our observed absence of SAGA at constitutive promoters (Fig. 2A,B). Both observations are in contrast to a prior study (Baptista et al. 2017), which we address in the Materials and Methods and Supplemental Figure S5, B–D.

The poised promoter class showed less SAGA dependency than induced promoters (Fig. 3; Supplemental Fig. S3A) despite having similar levels of SAGA (Fig. 2A). Since they nonetheless contain low levels of TBP and TAFs, similar to what is observed at constitutive promoters, we surmise that poised promoters, when in their poised state, have “constitutive-like” PICs that on average depend less on SAGA for PIC assembly than induced promoters (but more than constitutive promoters). Similar results and conclusions were drawn by examining steady-state or nascent mRNA (Supplemental Fig. S5B,C) and Pol II occupancy (Supplemental Fig. S5D). Therefore, SAGA and its recruitment by ssTFs are of central importance for augmented PIC assembly primarily at induced promoters, with a lesser contribution when promoters are poised. Little or no direct contribution is made at constitutive promoters.

### SAGA assembles a PIC that stabilizes TAF binding

For purposes of consistency with prior studies (Moqtaderi et al. 1996; Kuras et al. 2000; Li et al. 2000, 2002; Huisinga and Pugh 2004; Warfield et al. 2017; Petrenko et al. 2019), we examined the TFIID-dependency of PIC assembly. As expected, RP and constitutive (M04 and H04) promoters were TAF_dep_ (loss of TBP when Taf1 was depleted), while the induced (M02a and H02a) class was apparently TAF_ind_ (Fig. 3). Trends for these promoter classes were further confirmed through transcriptome analysis with a *taf1*^*ts2*^ mutant (Supplemental Fig. S5B). However, additional control experiments described below cast doubt on this generally accepted principle of TAF independence and drove us to reconsider the relationship between SAGA and TFIID in PIC assembly.

**Figure 4.**
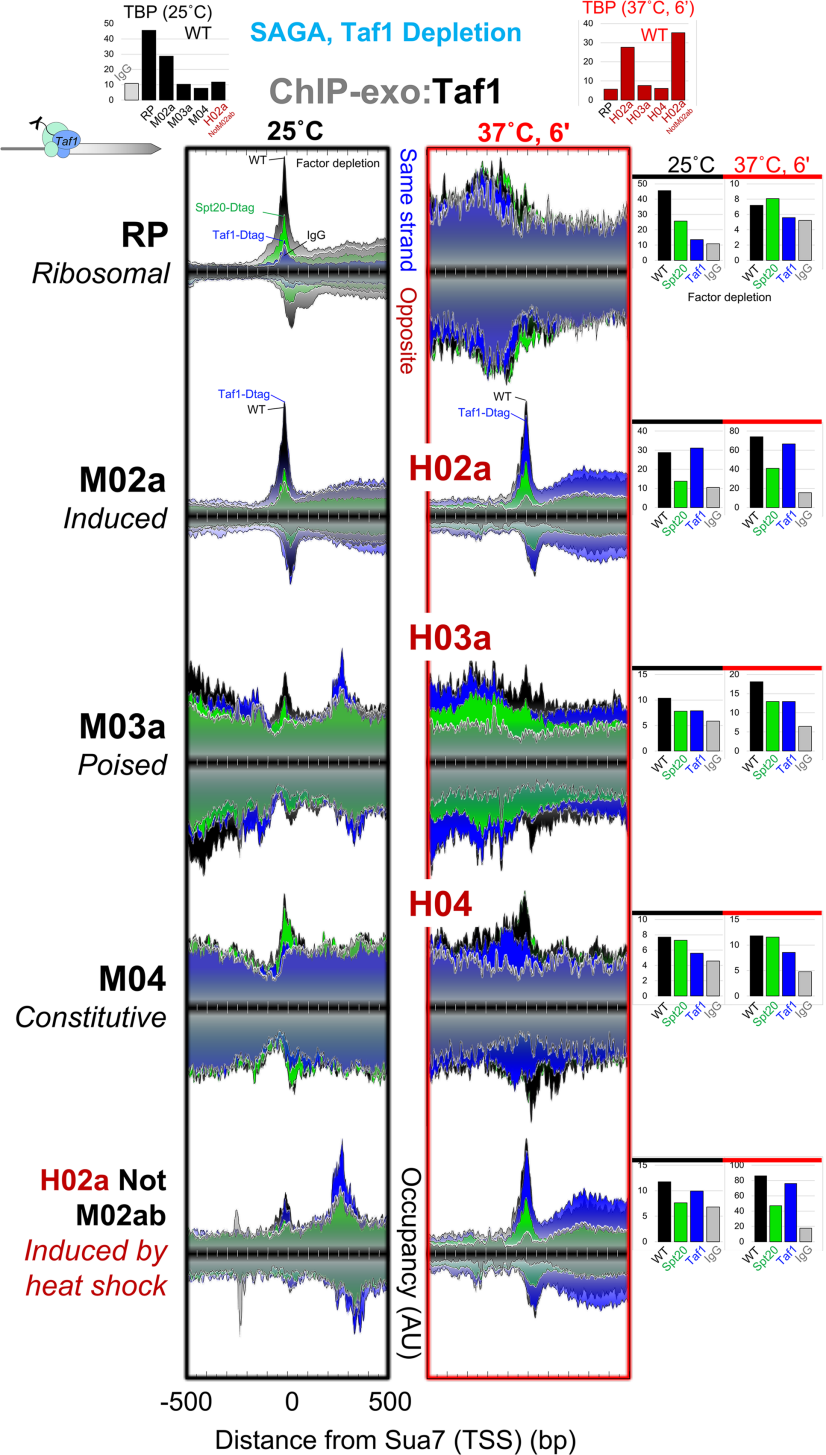
SAGA assembles a PIC that stabilizes TAF binding. Composite plots are shown of ChIP-exo tag 5′ ends of TFIID (Taf1) upon SAGA (Spt20) or Taf1 depletion, distributed around the Sua7 reference point within each promoter class at the indicated cellular temperature (25°C; 6 min at 37°C). See Figure 1 and the Materials and Methods for plotting details (analysis ID: CM43 and CM44).

When Taf1 was depleted by anchor-away and auxin-induced degradation, we monitored actual Taf1 occupancy loss at each promoter class. As expected, Taf1 was lost at RP, poised (M03a and H03a), and constitutive (M04 and H04) promoter classes (Fig. 4). Surprisingly, Taf1 was not lost at the induced promoter class, whether under normal (M02a) or heat shock (H02a) conditions. However, Taf1 could be depleted at these induced classes upon depletion of SAGA (Spt20). Thus, while TFIID (Taf1) is dynamic and depletable at most promoters, it stably associates with induced promoters in a SAGA-dependent manner.

This result was surprising, since pertinent models for induced promoters have SAGA assembling PICs either alone (TFIID/TAF_ind_) or redundantly with TFIID (Moqtaderi et al. 1996; Kuras et al. 2000; Li et al. 2000, 2002; Huisinga and Pugh 2004, 2007; Petrenko et al. 2019; Donczew et al. 2020). However, the results presented here indicate that prior depletion studies may not have actually depleted TAFs from induced promoters, despite their loss from all other promoters. SAGA and TFIID may be interdependent in PIC assembly at induced promoters, rather than completely independent or redundant. We suggest that SAGA drives PIC assembly at induced promoters, and this stably engages TFIID.

### SAGA acetylates +1 nucleosomes at inducible promoters, but non-SAGA Gcn5 acetylates constitutive promoters

We next examined H3K9 acetylation dependency on Gcn5. Gcn5 exists as part of at least two complexes: SAGA/SLIK and ADA (Grant et al. 1997; Sterner et al. 1999). Like other SAGA subunits, Gcn5 was enriched in the upstream region of inducible promoters (Fig. 2A), and its presence was dependent on SAGA (Spt20) (Supplemental Fig. S6).

**Figure 5.**
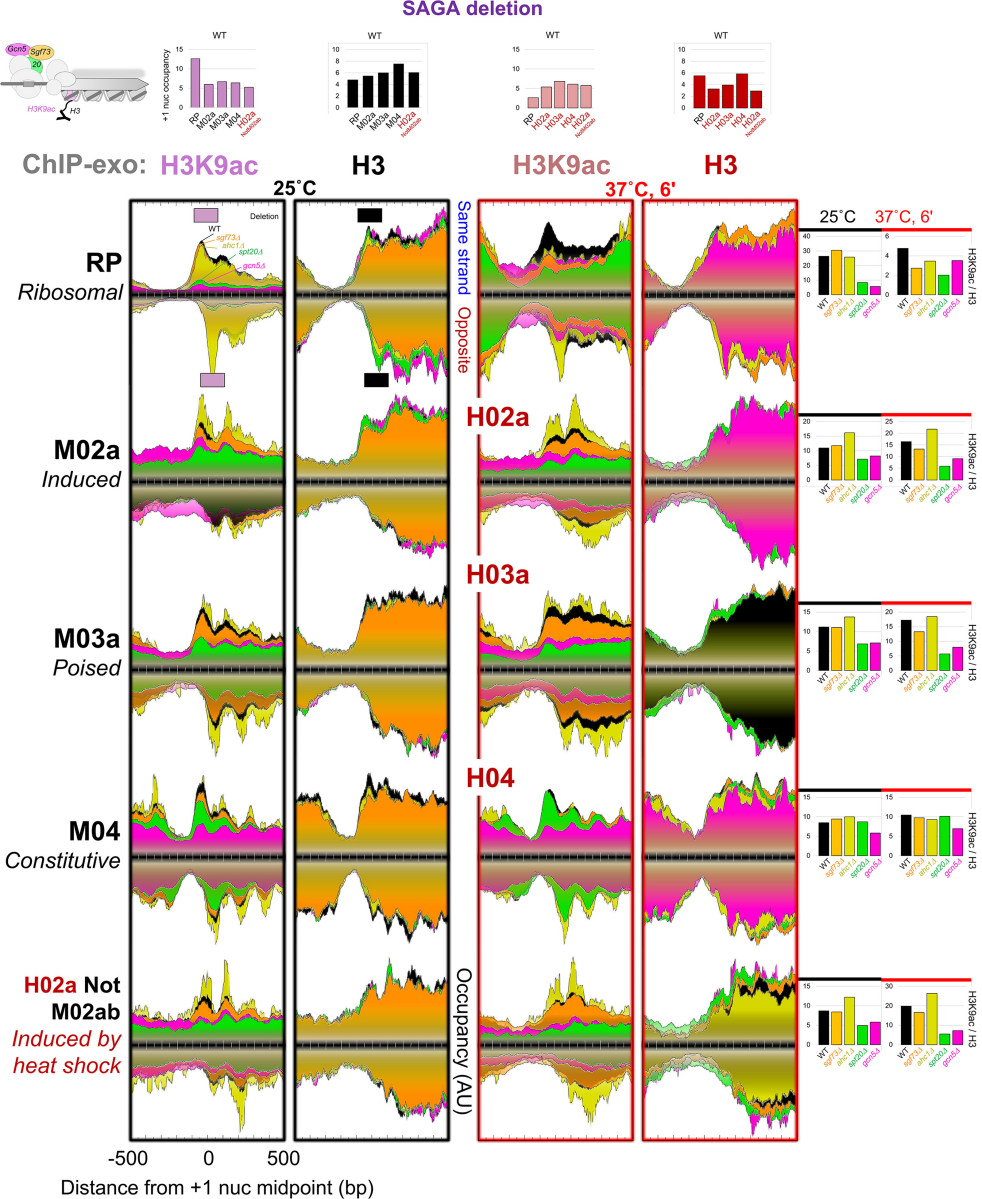
SAGA acetylates +1 nucleosomes at inducible promoters, whereas non-SAGA Gcn5 acetylates constitutive promoters. Composite plots are shown of ChIP-exo tag 5′ ends of H3K9ac and H3 upon deletion of SAGA (*sgf73*Δ, *spt20*Δ, and *gcn5*Δ) or ADA (*ahc1*Δ) subunits, distributed around the +1 nucleosome midpoint within each promoter class at the indicated cellular temperature (25°C; 6 min at 37°C). See Figure 1 and the Materials and Methods for plotting details. Quantification bar graphs at the *right* show H3K9ac/H3 density ratios at +1 nucleosomes assessed from −100 to +50 on the top strand and −50 to +100 on the bottom strand (see example horizonal boxes in RP composite plots). See also Supplemental Figure S7 (analysis ID: CM102, CM105, CM108, and CM111).

H3K9ac predominates at +1 nucleosome positions, with lesser acetylation at +2 (Zhang et al. 2011; Rhee et al. 2014). We examined H3K9ac density as an H3K9ac/H3 ratio, thereby controlling for any underlying changes in nucleosome occupancy. At all promoter classes, either deleting *GCN5* (*gcn5Δ*) or depleting Gcn5 at 25°C resulted in a substantial loss of H3K9ac density, whether assayed under normal or heat shock conditions (Fig. 5, yellow; Supplemental Fig. S7). This confirms prior genome-wide observations at 25°C (Bonnet et al. 2014). Minimal effects were observed on nucleosome (H3) occupancy, except where there were changes in gene expression. The residual H3K9ac density that remained after Gcn5 removal was equivalent to the background at other gene body nucleosome positions. Thus, under multiple cell states, Gcn5 acetylates H3K9 predominantly on the +1 nucleosome (and partially at +2) of all analyzed promoter classes.

In distinguishing the role of SAGA and ADA in H3K9 acetylation, we used *GCN5* deletion and Gcn5 depletion outcomes as a reference for maximal expected loss of H3K9ac with other SAGA or ADA subunits. Accordingly, loss of the ADA-specific subunit Ahc1 (Grant et al. 1997; Eberharter et al. 1999) resulted in increased H3K9ac density predominantly at inducible promoters (but not RP) and not at constitutive promoters (Fig. 5). This occurred under normal and heat shock conditions. ADA, as defined by Ahc1, may therefore be less active (or more repressive) for H3K9 acetylation in vivo than other Gcn5-containing complexes.

Deletion or depletion of SAGA-specific Spt20 resulted in loss of H3K9ac density predominantly at inducible promoters (RP, induced, and poised) (Fig. 5, green; Supplemental Fig. S7). This occurred under normal and heat shock conditions. The extent of H3K9ac loss was similar to that seen upon loss of Gcn5. Little or no loss was observed at constitutive promoters. Thus, SAGA acetylates +1 nucleosomes at all classes of inducible promoters but not at constitutive promoters. Gcn5 apart from SAGA or ADA acetylates constitutive promoters.

### SAGA deubiquitylase activity is specific to inducible promoters

**Figure 6.**
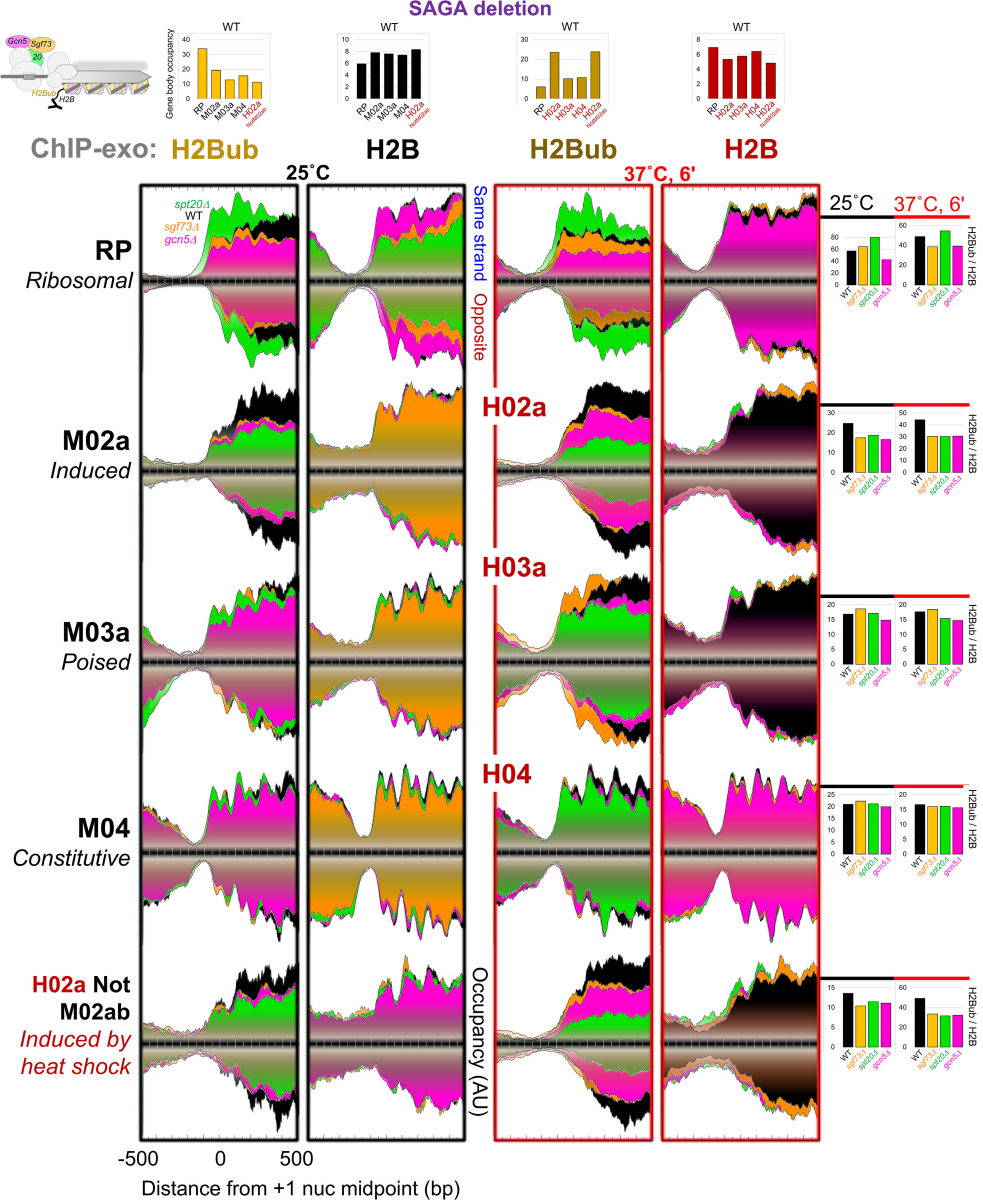
SAGA deubiquitylase activity is specific to inducible promoters. Composite plots of ChIP-exo tag 5′ ends of H2Bub and H2B upon deletion of SAGA (*sgf73*Δ, *spt20*Δ, and *gcn5*Δ) subunits, distributed around the +1 nucleosome midpoint within each promoter class at the indicated cellular temperature (25°C; 6 min at 37°C). See Figure 1 and the Materials and Methods for plotting details. Quantification bar graphs at the *right* show H2Bub/H2B density ratios at +1 nucleosomes assessed from −100 to +500 on the top strand, and −50 to +500 on the bottom strand (see example horizonal boxes in RP composite plots; analysis ID: CM113–116).

We next examined SAGA's H2B deubiquitylation activity at the five promoter classes. H2Bub predominates at genic nucleosome positions +1 through +4 (Batta et al. 2011; Bonnet et al. 2014; Gallego et al. 2020). As a transcription-linked mark, H2Bub levels are expected to track with PIC and nascent transcription levels, whereas the inverse is expected of H2B levels. Both trends were observed under normal and heat shock conditions (Fig. 6, top bar graphs). A recurring cycle of ubiquitylation and deubiquitylation is necessary for transcription (Henry et al. 2003). Any loss of transcription due to loss of SAGA (*spt20Δ, sgf73Δ*, and *gcn5Δ*) might stall the cycle at any point, resulting in elevated or depressed H2Bub levels. We observed this among the inducible promoter classes. For example, RP promoters showed increased H2Bub, whereas induced promoters showed decreased H2Bub in an *spt20Δ* strain. At constitutive promoters, loss of SAGA had little or no impact on H2B deubiquitylation under normal or heat shock conditions, reaffirming the lack of SAGA involvement at constitutive promoters.

## Discussion

### Most promoters are constitutive, having a simple SAGA-independent PIC architecture

**Figure 7.**
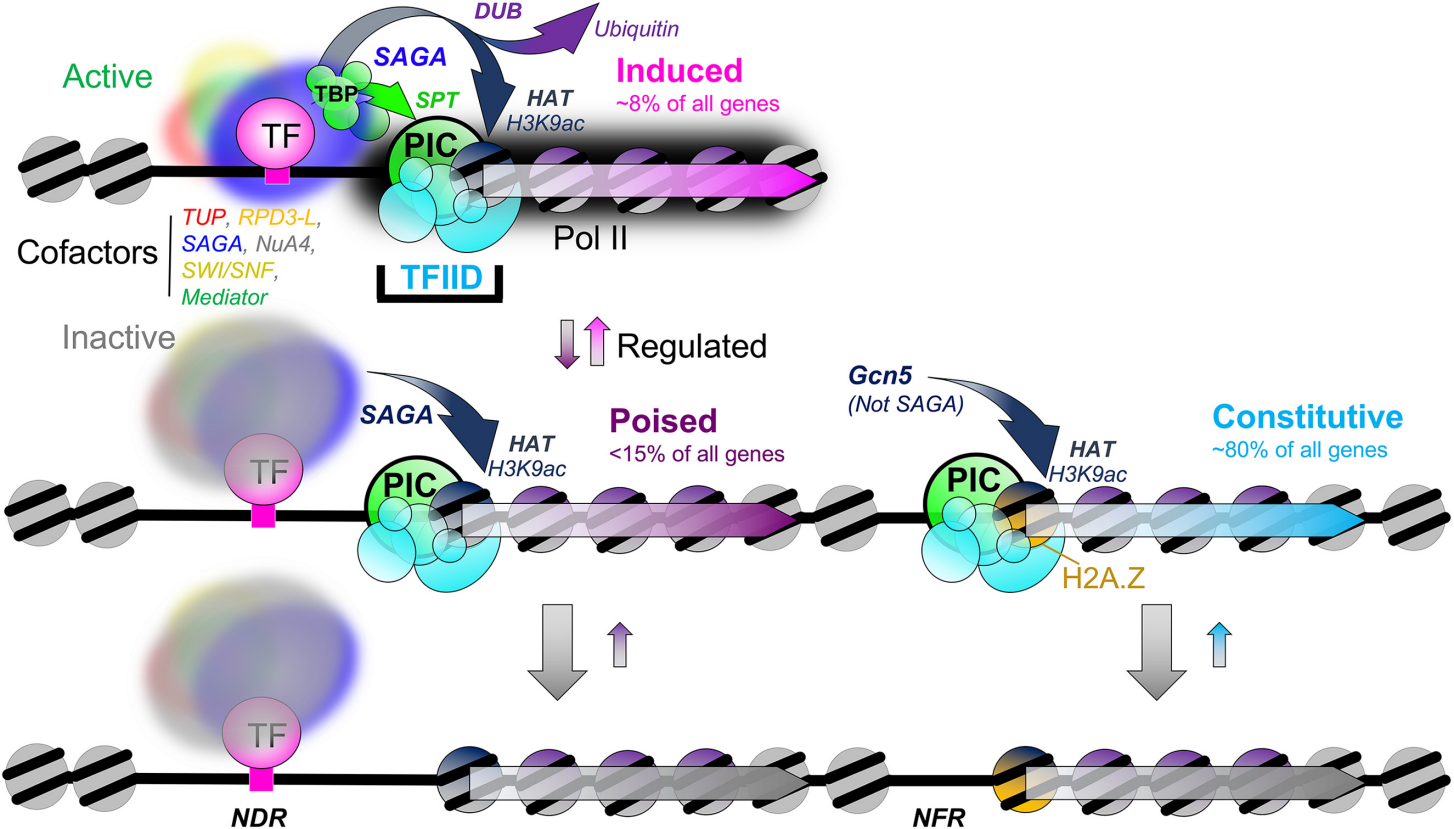
Model of PIC assembly at induced, poised, and constitutive promoters. (*Top left*) At induced promoters, ssTFs anchor activated cofactors such as SAGA that promote PIC assembly through TBP and H3K9 acetylation, which then locks in the TAF/TFIID complex. (*Middle* and *bottom left*) Poised promoters have essentially the same architecture, except the cofactors are relatively inactive for augmented PIC assembly. (*Right*) Constitutive promoters lack the inducible architecture and so depend predominantly on TFIID for PIC assembly. They use Gcn5 apart from SAGA to acetylate +1 nucleosomes (analysis ID: CM900).

Constitutive and inducible genes have distinct promoter architectures (Fig. 7, left vs. right side; Tirosh and Barkai 2008; Rossi et al. 2021). About 80% of all promoters are constitutive. They have well-organized nucleosome positions and high levels of H2A.Z and transcription-linked histone modifications (relative to PIC levels) that define an NFR only large enough to accommodate up to two divergent PICs. They do not contain, nor can their nucleosome organization accommodate, a complex containing a UAS, ssTFs, and their cofactors. In particular, we showed here that constitutive promoters do not directly depend on the ssTF-engaging SAGA cofactor for augmented TBP delivery, H3K9 acetylation, and H2B deubiquitylation. However, Gcn5 apart from SAGA provides H3K9ac at constitutive promoters. PIC assembly at constitutive promoters is infrequent (Nguyen et al. 2021), and this is reflected in the properties of TFIID rather than TAF_ind_ TBP. These properties, along with enrichment of less-studied proteins such as Bye1, characterize the architecture of a constitutive promoter. While constitutive promoters largely have a simple protein architecture that can include sequence-specific insulation from upstream events, a more granular analysis would be needed to determine whether additional architectural subclasses exist.

### Inducible promoters are largely poised with latent ssTFs and cofactors

About 20% of all promoters are inducible. Their function is to sense environmental signals and create a greater dynamic range of expression compared with constitutive promoters. The inducible class tends to have a UAS that anchors ssTFs and multiple cofactors (e.g., SAGA, Mediator, TUP, Nua4, SWI/SNF, and RPD3-L) to the promoter region (Rossi et al. 2021). Consequently, their chromatin architecture includes an NDR that extends hundreds of base pairs upstream of where the PIC assembles. Inducible promoters can be classified according to whether they are induced, poised, or repressed in a particular condition. Most inducible promoters are in a poised state. The protein architecture in the poised state includes ssTFs and the main cofactors tethered to a UAS, but they are not particularly active in augmenting PIC assembly. However, in the poised state, SAGA contributes to +1 nucleosome acetylation, which contributes to low levels of PIC assembly. Poised promoters, like induced promoters, are largely histone-depleted at the UAS, meaning that nucleosomes and any altered or subnucleosomal structures are generally absent.

The ssTFs at poised promoters differ from those at induced promoters. This may reflect ssTFs at induced promoters being activated by existing environmental signals, whereas the ssTFs at poised promoters may be tuned to a different set of signals that are not present. The notion of a poised state makes sense in light of a rapid response to acute changes in environmental conditions. Induced promoters appear much like poised promoters, except their cofactors actively augment PIC assembly, which results in high levels of gene expression.

Since we had not demonstrated that the poised promoter class can in fact be induced, we also examined a set of condition-specific heat shock promoters (algorithm ID: H02aNotM02ab). They are quiescent at 25°C but are rapidly and robustly induced by 6 min of heat shock (37°C). Their induction is defined as having high GTF:TAF (Sua7:Taf2) ratios at 37°C but not at 25°C. Like poised promoters, we found these condition-specific promoters to have the main complement of ssTFs and cofactors at the UAS both before and after induction.

ssTFs such as Hsf1 and Gal4 do not require inducing signals to bind their cognate site(s) (Sorger and Pelham 1988; Rhee and Pugh 2011; Rossi et al. 2021), although such signals do enhance binding. We found that Hsf1 binding to its cognate DNA site occurs independent of SAGA. However, Hsf1/UAS binding is required to recruit SAGA (and presumably other cofactors). Other ssTFs may be sequestered in the cytoplasm and only activated to translocate into the nucleus and bind DNA by specific signals. They would not be expected to participate in the poised state. Some uninduced promoters have bound ssTFs but are not occupied by the main cofactors. For example, in glucose (repressive) media, the GAL1,10,7 gene cluster is not in a poised state, as it lacks the major cofactors (Lue et al. 1987; Ma and Ptashne 1987; Rossi et al. 2021). Instead, it contains the Gal4/Gal80-repressive complex.

With ssTFs/cofactors already present in the NDR, the historical view of gene activation in budding yeast may be expanded. At some promoters, certain ssTFs might invade and evict nucleosomes while recruiting cofactors and the PIC. However, at most other inducible promoters, ssTF and cofactors are generally present, and resident nucleosomes are already depleted. They proceed through a measurable state change that augments the recruitment of PIC components. This is manifested by a locally broadened ChIP-exo pattern for cofactors. ssTFs/cofactors, whether latent or active, generally do not stably engage GTFs, TAFs, or Pol II, as we see little cross-linking at the UAS for these PIC components compared with ssTFs and cofactors. However, transient interactions are not ruled out. Rap1 is an exception in having measurable TAF interactions at the UASs of RP promoters. Reciprocally, we saw little or no cross-linking of ssTFs and cofactors at core promoters like we saw for PIC components. Thus, unlike the main cofactors (SAGA, Mediator, TUP, NuA4, SWI/SNF, and RPD3-L), PIC components are not stably tethered and poised at the UAS, waiting to be deposited upon induction. Reciprocally, the main cofactors are not stably engaged with the PIC.

### How a TAF complex might be recruited to promoters

Our data suggest that yeast promoters generally have a single TFIID-containing PIC position. TFIID may have evolved to be intrinsically inefficient in PIC assembly, which is manifested at constitutive and poised promoters. There, Taf1 may inhibit TBP/DNA interactions and may have weak interactions with acetylated +1 nucleosomes (Bhuiyan and Timmers 2019). The weaker TBP/DNA interactions afforded by the TATA-less nature of constitutive promoters, along with TFIID ejection during initiation (Knoll et al. 2020), add to this inefficiency. This combined low-affinity architecture may be sufficient to provide low levels of PIC assembly (∼0.3% of what is seen at tRNA promoters) and low levels of constitutive transcription (Nguyen et al. 2021). By this mechanism, TBP is recruited to promoters via the TAF complex (Li et al. 2000), and so GTF occupancy is limited by the presence of the TAF complex. This results in an observed GTF:TAF occupancy ratio of ∼1.

At induced promoters, two alternative high-efficiency TAF recruitment mechanisms may be in play in addition to the low-affinity interaction that is characteristic of constitutive promoters. One high-efficiency mechanism is exemplified at RP promoters, although a limited number of other induced promoters that use a similar mechanism is not ruled out. At RP promoters, Rap1 directly engages with TAFs and TFIIA in a high-affinity but dynamic interaction that may eliminate the inefficiencies inherent at constitutive promoters and allows for high PIC assembly (Garbett et al. 2007; Layer et al. 2010; Papai et al. 2010; Layer and Weil 2013; Reja et al. 2015). In support of this, Rap1–TAF interactions in vivo do not require TBP (Li et al. 2000). By this mechanism, TBP and TFIIA are recruited to promoters via the TAF complex, thereby limiting it to a GTF:TAF ratio of ∼1.

In the second high-efficiency TAF recruitment mechanism, ssTFs (but not Rap1) and SAGA deliver TBP to the TATA-box at induced promoters to form a PIC. This is a stable interaction of TBP with DNA that supports transcriptional bursting. Consequently, and unlike the other mechanisms, TBP binds DNA first and then engages the TAF complex, possibly through TFIIA (Patel et al. 2018). This allows for a higher GTF:TAF ratio, since an initial PIC could exist without the TAF complex. TAF binding at this class of induced promoters is therefore SAGA-dependent, as we observed here. Higher PIC occupancy would also result in more TFIID (Taf1) binding, as exemplified at the heat shock-induced class. Whether transcription takes place at these induced promoters in the absence of TAFs is unclear. However, prior studies reporting an apparent TAF independence may not have actually removed TAFs from these promoters (Kuras et al. 2000; Li et al. 2000, 2002; Huisinga and Pugh 2004).

### SAGA is a gene-specific cofactor

How SAGA contributes to transcriptional regulation has been the subject of much debate. Early studies showed that SAGA contributed to the expression of highly regulated genes, with minor contributions at most genes (Huisinga and Pugh 2004), which are constitutive. Later evidence suggested that SAGA was a general cofactor at all genes (Baptista et al. 2017), although follow-up studies placed qualifications on this assertion (Donczew et al. 2020), including in human systems (Fischer et al. 2022). Our follow-up assessment adds more qualifications (see the Materials and Methods). Here, we report that certain SAGA activities are highly selective for induced promoter states in augmenting PIC assembly, stabilizing TAF binding, and effecting H2B deubiquitylation. These results indicate that SAGA and TFIID are intertwined in PIC assembly when promoters are induced. SAGA is more broadly limited to inducible promoters (RP, induced, poised, and condition-specific) for UAS binding and for H3K9 acetylation of +1 nucleosomes. All other promoter +1 nucleosomes are acetylated via a SAGA-independent Gcn5. This entity may not be ADA as defined here by Ahc1 function (Eberharter et al. 1999), but this possibility or potential variants are not excluded given the wide variety of Gcn5-containing complexes in eukaryotes (Soffers et al. 2019; Soffers and Workman 2020). SAGA may indirectly make some contribution to the transcription of all genes by regulating inducible genes that subsequently have global effects on transcription.

Our study suggests a unifying principle whereby essentially all Pol II promoters contain a TFIID-anchored PIC. This might allow inducible and constitutive promoters to evolutionarily interconvert through a gain or loss of a UAS, along with adjustments to core promoter sequences such as the TATA-box (and likely other regions). Inducible regulatory mechanisms are outfitted with multiple regulatable molecular switches, including environment-sensing ssTFs, nucleosome remodeling, and PIC assembly. The same switches are well established in multicellular eukaryotes, including humans, and so the intertwined mechanisms of constitutive and inducible PIC assembly described here are likely to be applicable in other eukaryotic systems.

## Materials and methods

### Availability of materials

Further information and requests for reagents may be directed to and will be fulfilled by B.F.P. (fp265@cornell.edu).

### Cell growth

*S. cerevisiae* cultures were grown in 50 mL of YPDA medium to an OD_600_ of 0.6–0.8 at 25°C. Formaldehyde at a final concentration of 1% (v/v) was used to cross-link cells for 15 min at room temperature, and 125 mM glycine was used to quench for 5 min. For inducing acute heat shock at 37°C, 50 mL of YPDA at 55°C was added to 50 mL of cultures and quickly transferred to a shaker incubator (Badjatia et al. 2021) for 6 min at 37°C. Maximal heat shock effects on PIC assembly were achieved by 6 min (Vinayachandran et al. 2018). Cells were then cross-linked with chilled formaldehyde for 15 min in a 25°C shaker followed by quenching with glycine for 5 min. Cross-linked cells were centrifuged at 4000 rpm for 5 min at 4°C and washed with ST buffer (10 mM Tris-HCl at pH 7.5, 100 mM NaCl). Supernatants were removed, and cell pellets were flash-frozen and stored at −80°C until further use. Tallies of independent biological replicates are reported in Supplemental Table S4. TAP-tagged strains in BY4741 background were purchased from Open Biosystems. D-tagged strains (see below) were constructed in a modified W303 background. Hsf1-FRB and corresponding parental strains were in BY4742 background and were a gift from Dr. David Gross (Louisiana State University).

### Imaging of D-tagged yeast cells

Exponentially growing yeast cells were immobilized on microscope slides with agarose pads and imaged on a Leica fluorescent microscope (Leica DMI6000). Images were acquired and recorded with a Hamamatsu ORCA-R2 C10600 camera. Fluorescence and phased-contrast images were captured and processed using developed Matlab software. To deplete D-tagged factors from the nucleus, cells immobilized on agarose pads were incubated with 1 μg/mL (final) rapamycin for 60 min at room temperature. Changes in fluorescence were subsequently measured and recorded.

### Plate growth assays

Exponentially growing D-tagged and corresponding parental strains were serially diluted 10-fold, and 5 μL of cultures was spotted on YPDA agar plates containing 1 mM (final) auxin or 1μg/mL rapamycin alone or in combination. Plates were incubated for 2–3 d at 30°C.

### Dual depletion/degradation system

Depletion was achieved using a combined anchor-away (AA) and auxin-induced degradation (AID) system in which a D tag was fused to the C terminus of the factor to be removed (Supplemental Fig. S2A; Badjatia et al. 2021). This offered maximum responsiveness and flexibility for depletion. Due to the presence of both AA and AID epitopes, tagged factors were depleted and simultaneously degraded using 1 μg/mL (final) rapamycin and 1 mM indole-3-acetic acid for 30 min (except for Hsf1 depletion, which contained only the FRB tag and thus used only rapamycin). These strains are similar to the ones described previously (Badjatia et al. 2021), except they lacked the L tag. yAB001 was used as a parental control strain, which was used to fuse D tags to the *SPT20, SPT7*, *GCN5, TRA1, TAF1*, and *SUA7* genes.

### Cell lysis and chromatin shearing

Fifty milliliters of cell pellets was resuspended in 750 μL of FA lysis buffer (50 mM HEPES-KOH at pH 7.5, 150 mM NaCl, 2 mM EDTA, 1% Triton X-100, 0.1% sodium deoxycholate, complete protease inhibitor [Roche]) along with 1 mL of 0.5-mm acid-washed zirconia beads. Lysis was performed by bead beating for four cycles of 3 min on and 7 min off in a Mini-Beadbeater-96 machine (Biospec). Whole-cell lysates were centrifuged at 14,000 rpm for 3 min at 4°C. Cytoplasmic supernatants were discarded, and nuclear pellets were resuspended in 200 μL of FA lysis buffer supplemented with 0.1% SDS and 75 μL of 0.1-mm zirconia beads. Nuclear samples were sonicated in a Bioruptor (Diagenode) for four cycles of 30 sec on and 30 sec off. Samples were centrifuged at 14,000 rpm for 10 min to collect sonicated chromatin fraction, which was predominantly enriched with DNA fragments in a range of 100–500 bp in size (verified on agarose gels).

### Antibodies

The following antibodies were used in ChIP-exo: anti H3K120ub (Cell Signaling 5546S), anti-H2B (Abcam 1790), nonspecific IgG (Sigma i5006), anti-TBP (Abcam 61411), anti-Pol II (Santa Cruz Biotechnology 8WG16), anti-H3 (Abcam 1791), anti-H3K9ac (Millipore Sigma 07-352), and anti-H3K14ac (Abcam ab52946). Sera against Hsf1, Taf1, and SAGA subunits (Gcn5, Sgf73, and Spt3) were obtained from David Gross (Louisiana State University), Joe Reese (Pennsylvania State University), and Tony Weil (Vanderbilt School of Medicine), respectively.

### ChIP-exo 5.0

Chromatin obtained from 50 mL of culture was used for a single ChIP experiment. Appropriate antibodies (3–5 μg per ChIP) were conjugated to protein A/G magnetic Sepharose resin (GE Healthcare Life Sciences) for 6–8 h at 4°C. Unconjugated antibody was removed, and then chromatin samples were incubated overnight (12–16 h) at 4°C with constant mixing. For TAP-tagged samples, 40 μL of IgG-Dynabead slurry was used per sample. Subsequent steps were performed as described previously (Rossi et al. 2018).

### DNA sequencing and bioinformatics

ChIP-exo libraries were gel-purified and sequenced in a paired-end mode with the NextSeq 500 (read_1 had 40 cycles, and read_2 had 36 cycles). Sequenced reads were aligned to the *S. cerevisiae* genome (sacCer3) using BWA-MEM (v.0.7.9a) (Li 2013). Aligned reads were filtered using Picard (http://broadinstitute.github.io/picard) and SAMtools (Li et al. 2009) to remove PCR duplicates (i.e., where the 5′ coordinate strands of read-1 and read-2 were identical to another read pair) and nonuniquely mapping reads. All data were normalized by NCIS (Liang and Keleş 2012), except for histone targets, which were NFR-normalized (essentially setting the sum of a 50-bp window centered on 5378 gene NFR midpoints to be equal across samples). NFR midpoints are defined in Rossi et al. (2021) and reported in Supplemental Table S1, column J.

### Data and code availability

Prior data sets used in this study can be accessed at NCBI Gene Expression Omnibus (GEO; https://www.ncbi.nlm.nih.gov/geo) with accession numbers GSE147927 (Rossi et al. 2021), GSE151348 (Badjatia et al. 2021), and GSE81127 (Jeronimo et al. 2016).

Data sets generated in this study can be accessed with accession number GSE212655 (GSE212653 for RNA, and GSE212654 for ChIP-exo).

Analysis was performed with CLI ScriptManager v.013 (https://github.com/CEGRcode/scriptmanager, doi:10.1145/3491418.3535161).

Additional information about data analysis, custom scripts, parameters, and curation are provided in detail on Github (https://github.com/CEGRcode/2022-Mittal_SAGA). Links to the up-to-date curated and interactive data will be available through the GitHub repo.

## Supplementary Material

Supplemental Material
