# Supplementary material for: An integrated SAGA and TFIID PIC assembly pathway selective for poised and induced promoters
Source: Genes Dev. 2022 Sep 1;36(17-18):985–1001. doi: 10.1101/gad.350026.122 (PMC9732905; doi:10.1101/gad.350026.122)
Supplement: Supplemental Material [file supp_36_17-18_985__DC1.html]

An integrated SAGA and TFIID PIC assembly pathway selective for poised and induced promoters — Supplemental Material 

# An integrated SAGA and TFIID PIC assembly pathway selective for poised and induced promoters

## Supplemental Material

- Supplemental\_Figures.pdf
- Supplemental\_Material\_Content.pdf
- Supplemental\_Methods.pdf
- Supplemental\_Table\_S1.xlsx
- Supplemental\_Table\_S2.xlsx
- Supplemental\_Table\_S3.xlsx
- Supplemental\_Table\_S4.xlsx
