# Supplementary material for: An integrated SAGA and TFIID PIC assembly pathway selective for poised and induced promoters
Source: Genes Dev. 2022 Sep 1;36(17-18):985–1001. doi: 10.1101/gad.350026.122 (PMC9732905; doi:10.1101/gad.350026.122)
Supplement: Supplemental Material [file supp_gad.350026.122_Supplemental_Figures_.pdf]

Supplemental Figures

Chitvan Mittal, Olivia Lang, William K.M. Lai, and B. Franklin Pugh (2022)
An integrated SAGA and TFIID PIC assembly pathway selective for poised and induced promoters

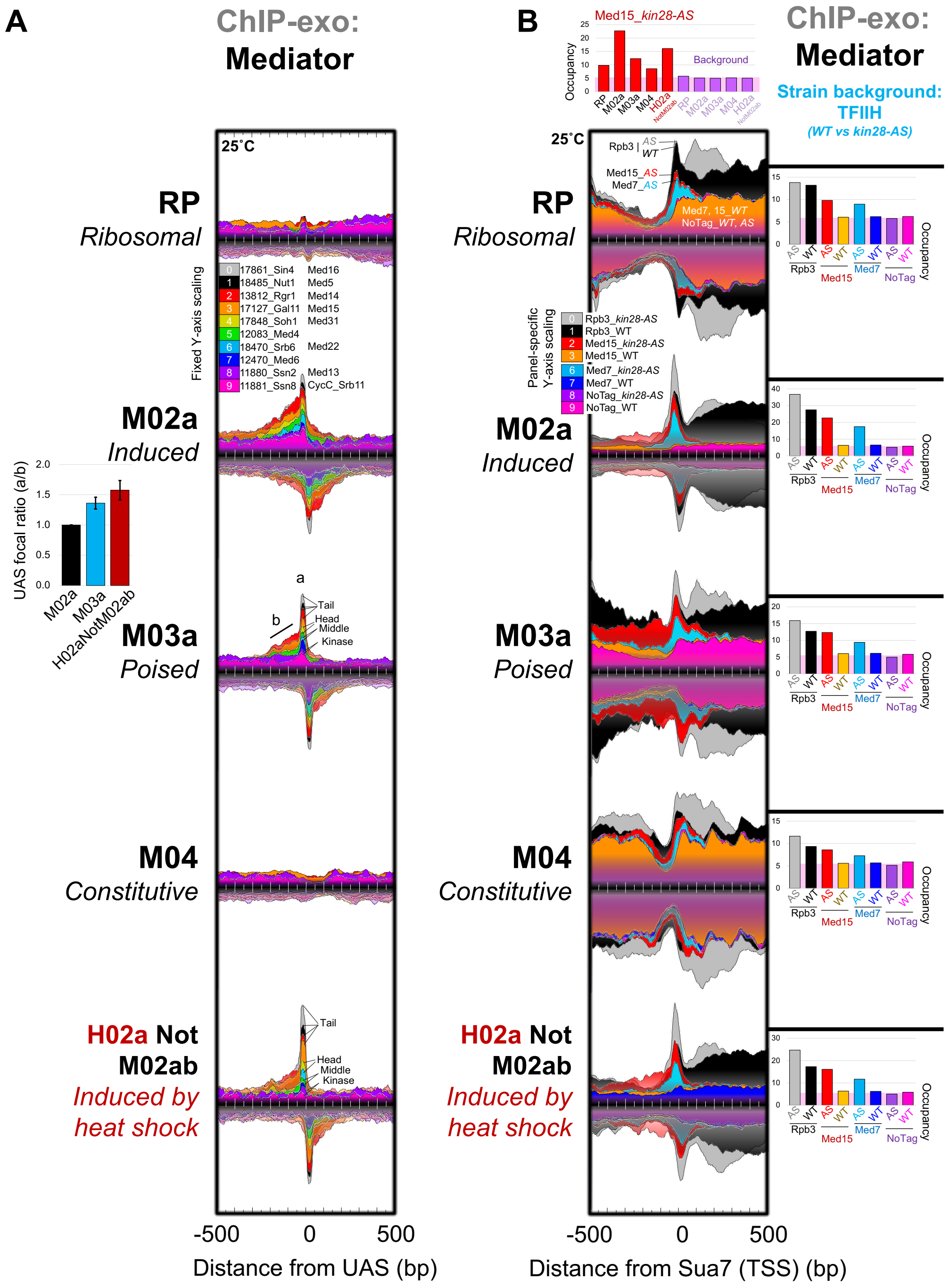

Supplemental Fig. S1. Distinct Mediator states at inducible and constitutive promoters. (*A*) Composite plots are shown of tag 5′ ends from ChIP-exo of indicated Mediator subunits distributed around the UAS (or equivalent) reference point within each promoter class. See Fig. 1 and Methods for plotting details. The y-axis occupancy scale is linear and the same for a particular target across the different promoter classes, and thus are comparable within a target. The bar graph to the left quantifies the UAS focal ratio, which is the area demarcated by "a" (occupancy from -30 to 0 on the TSS strand and from 0 to +30 on the opposite strand) divided by the area demarcated by "b" (occupancy from -200 to -30 from the UAS on the TSS strand and from +30 to +200 on the opposite strand), then averaged across head, middle, tail subunits of Mediator (i.e., all but Ssn2 and Ssn8). Related to Fig. 1. (analysis ID: CM708). (*B*) Composite plots are shown for the indicated Mediator subunits (Med 15, Med7) or Pol II (Rbp3) distributed around the Sua7 reference point within each promoter class in a wild type or kin28as mutant strain. Kin28as is inhibited by 6 μM 1-Naphthyl PP1 (NAPP1; analysis ID: CM70).

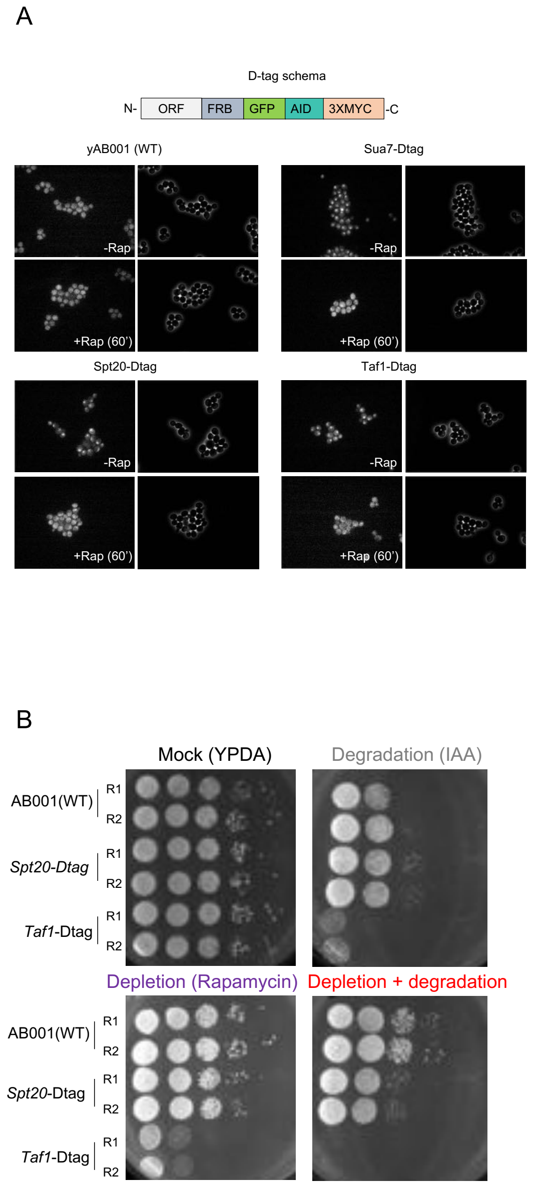

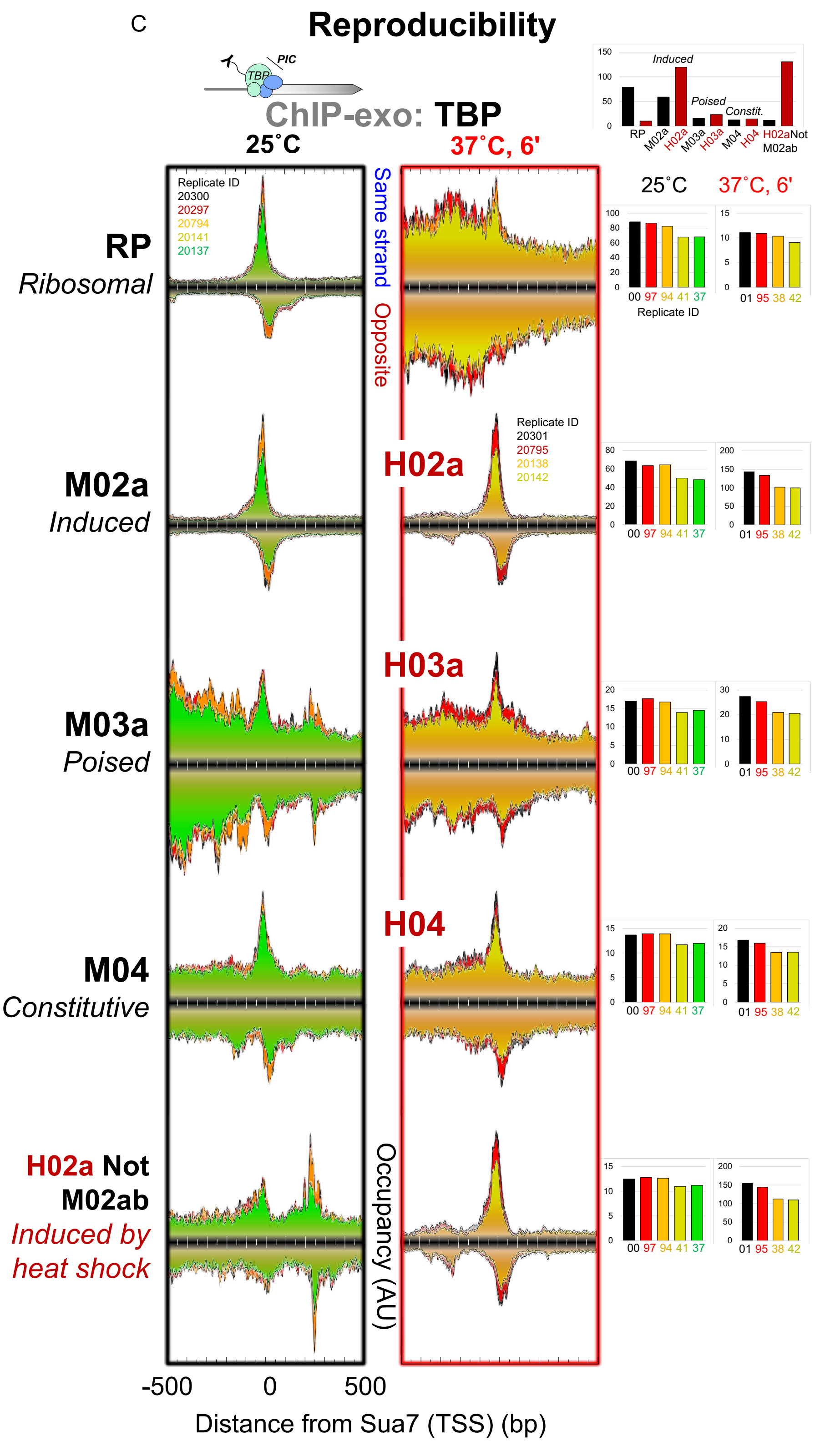

Supplemental Fig. S2. D-tagged strain properties and controls. (*A*) Strains in which the indicated depletion target contained the following: an FRB anchor-away tag, a GFP tag for monitoring nuclear-cytoplasmic translocation, an AID tag for auxin-induced degradation, and a myc tag (collectively a D-tag). yAB001 is the parent lacking a D-tag. All were grown to log phase (OD 0.8) and incubated with rapamycin (Rap, 1 ug/mL) for 60 minutes. For each set of four images, GFP fluorescence is monitored on the left, and phase contrast on the right. Upper subpanels lack rapamycin, whereas lower subpanels contain rapamycin. Punctate nuclear localization in the absence of rapamycin is observed to become more diffuse in the presence of rapamycin (lower panels; analysis ID: CM200). (*B*) Growth assays. The indicated strains were serially 10-fold diluted and spotted on agar YPD media plates containing 1 ug/ mL Rap (lower left), 1 mM IAA (upper right), both (lower right), or neither (upper left), and allowed to grow at 30 ˚C. (analysis ID: CM201). (*C*) Reproducibility of TBP ChIP-exo. Shown are biological replicates performed over the course of this project at the indicated cellular temperature (25 ˚C; 37 ˚C, 6'), along with their internal (PEGR) ID (analysis ID: CM39, CM41).

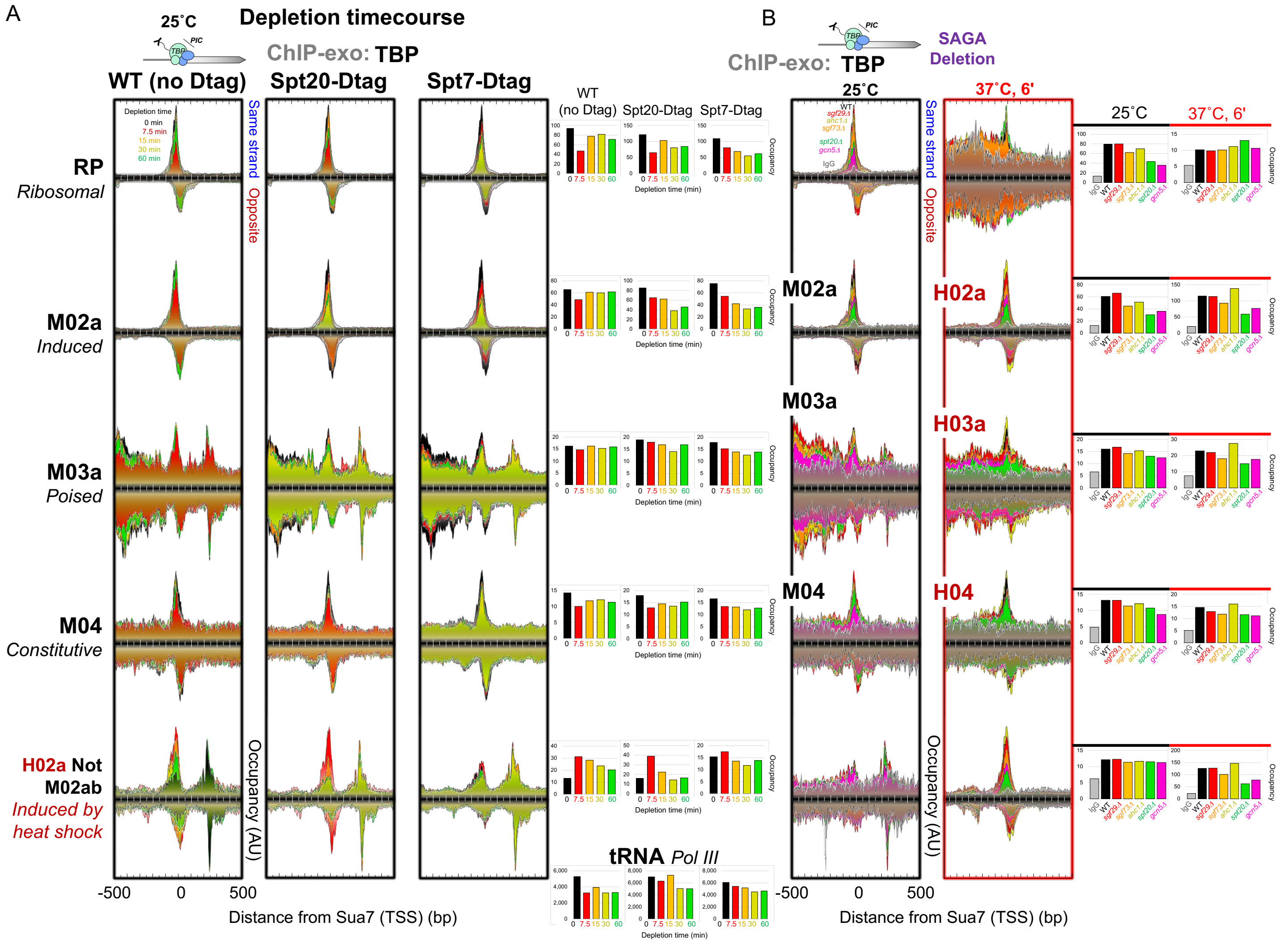

A
Depletion timecourse
25˚C
ChIP-exo: TBP
WT (no Dtag)
Spt20-Dtag
Spt7-Dtag
RP
Ribosomal
M02a
Induced
M03a
Poised
M04
Constitutive
H02a Not M02ab
Induced by heat shock
Same strand
Opposite
Occupancy (AU)
-500
0
500
Distance from Sua7 (TSS) (bp)
tRNA Pol III
Depletion time (min)
B
ChIP-exo: TBP
SAGA Deletion
25˚C
37˚C, 6'
H02a
H03a
H04

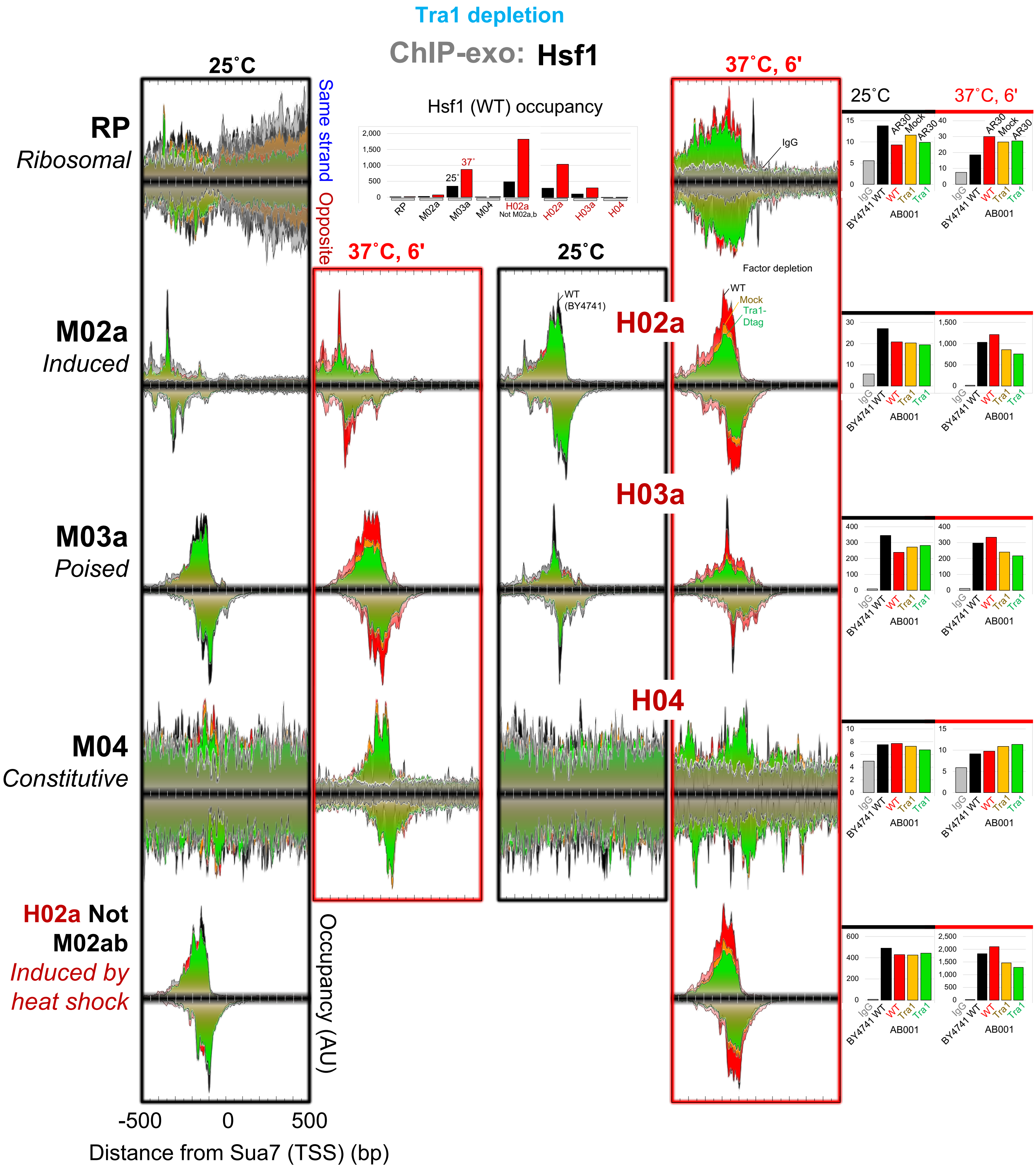

Supplemental Fig. S4. Stability of Hsf1 to depletion of Tra1. Tra1 was depleted at 25 ˚C for 30 min. then Hsf1 occupancy measured at the indicated cellular temperature (25 ˚C; 37 ˚C for 6 min.). Composite plots of tag 5' ends from ChIP-exo of Hsf1 are distributed around the Sua7 reference point within each promoter class. See Fig. 1 and Methods for plotting details. The y-axis occupancy scale is linear and the same within a promoter class for mutant and WT, and quantified in the bar graphs to the right (for M-series at 25 ˚C and H-series at 37 ˚C, 6 min). The y-axis occupancy is scaled separately among promoter classes to maximize visual distinction and thus are not directly comparable. Comparable values are provided in bar graph (top). Bar graph quantification is relative to the reference point (Sua7) from -225 to -75 on the top (TSS) strand plus from -200 to -50 on the opposite strand. The Hsf1 signal at M04 at 37 ˚C, 6 min is ~1% of that at H02aNotM02ab. Related to Fig. 2 (analysis ID: CM45, CM46).

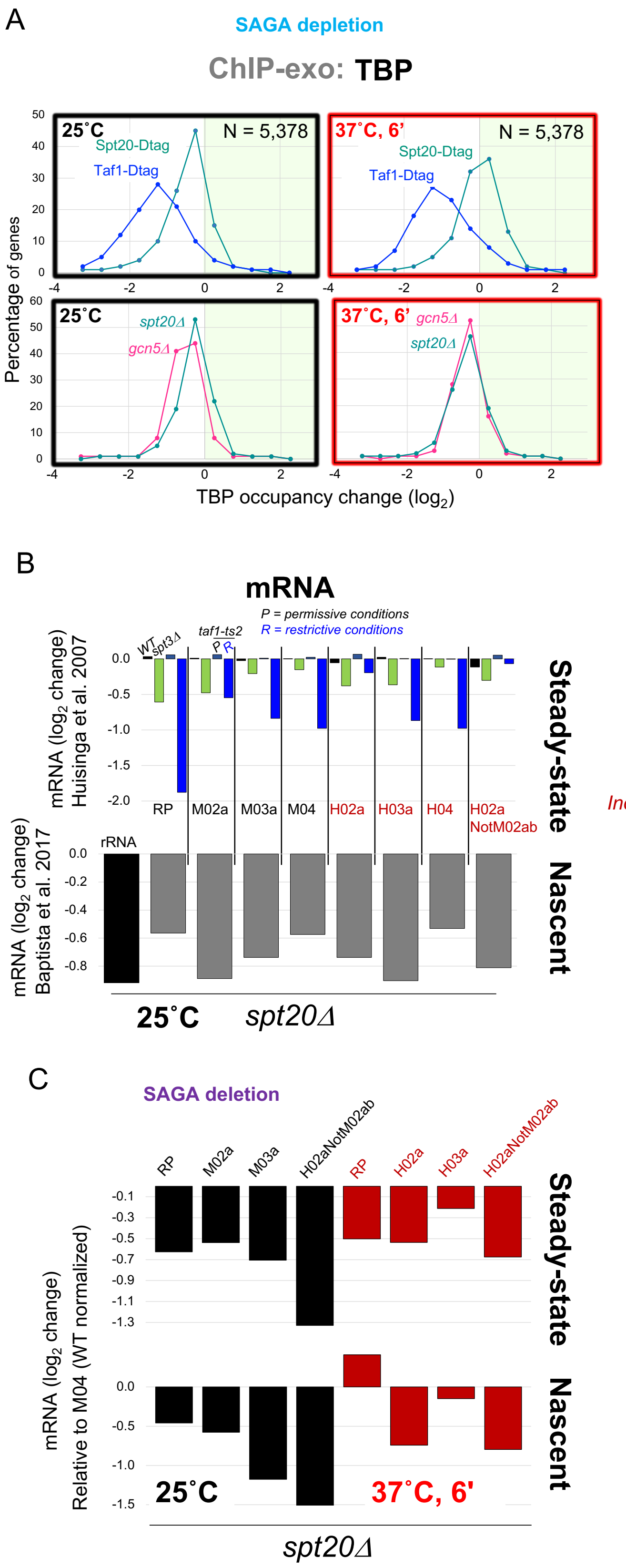

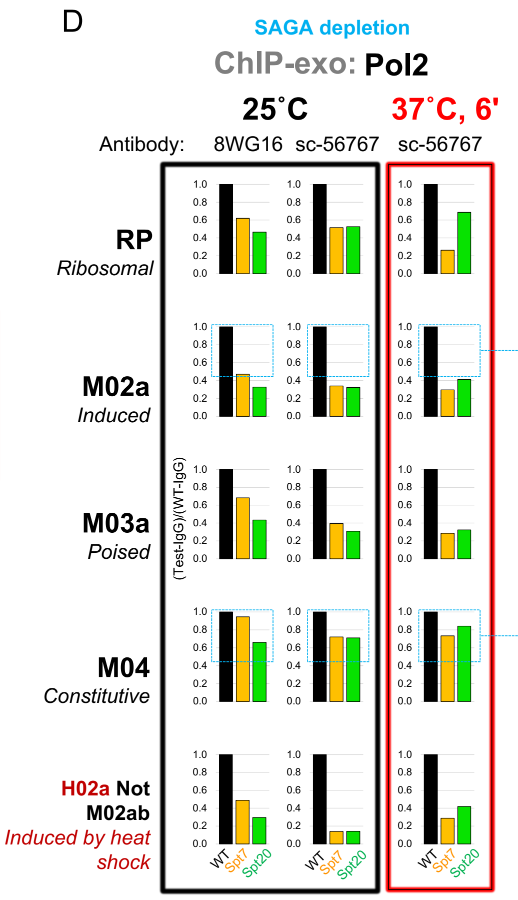

Supplemental Fig. S5. Orthogonal data indicating that SAGA primarily drives induced PIC assembly. Related to Fig. 3. (*A*) Frequency distribution of fold changes in TBP occupancy at individual promoters in the indicated mutants relative to a WT strain. 5,378 Pol II-transcribed promoters were examined. Changes were examined at the indicated cellular temperature (25 ˚C; 37 ˚C for 6 min.). (analysis ID: CM202). (*B*) Fold changes in mRNA levels in the indicated mutant strains relative to WT are plotted on a log_2 scale. Upper panel is steady-state data from (Huisinga and Pugh, 2004). Lower panel is 4-tU nascent mRNA data from (Baptista et al., 2017)analysis ID: CM203). (*C*) Fold changes in steady-state (upper) or nascent (4tU-labeling, lower) mRNA levels in a *spt20Δ* strains for the indicated promoter classes. Bar graph quantification is relative to the reference point (Sua7) from -70 to +500 on the top (TSS) strand plus from -40 to +500 on the opposite strand. Data are plotted on a log_2 scale relative to M04 (constitutive) at the indicated cellular temperature (25 ˚C; 37 ˚C for 6 min; analysis ID: CM500-503). (*D*) Pol II loss upon SAGA (Spt7 or Spt20) depletion. Bar graph quantification is relative to the reference point (Sua7) from -70 to +500 on the top (TSS) strand plus from -40 to +500 on the opposite strand. Changes were examined at the indicated cellular temperature (25 ˚C; 37 ˚C for 6 min.). Antibody catalog number for ChIP is indicated. Cyan boxes emphasize the relative occupancy difference in M02a vs M04 promoter classes (analysis ID: CM20, CM22, CM27).

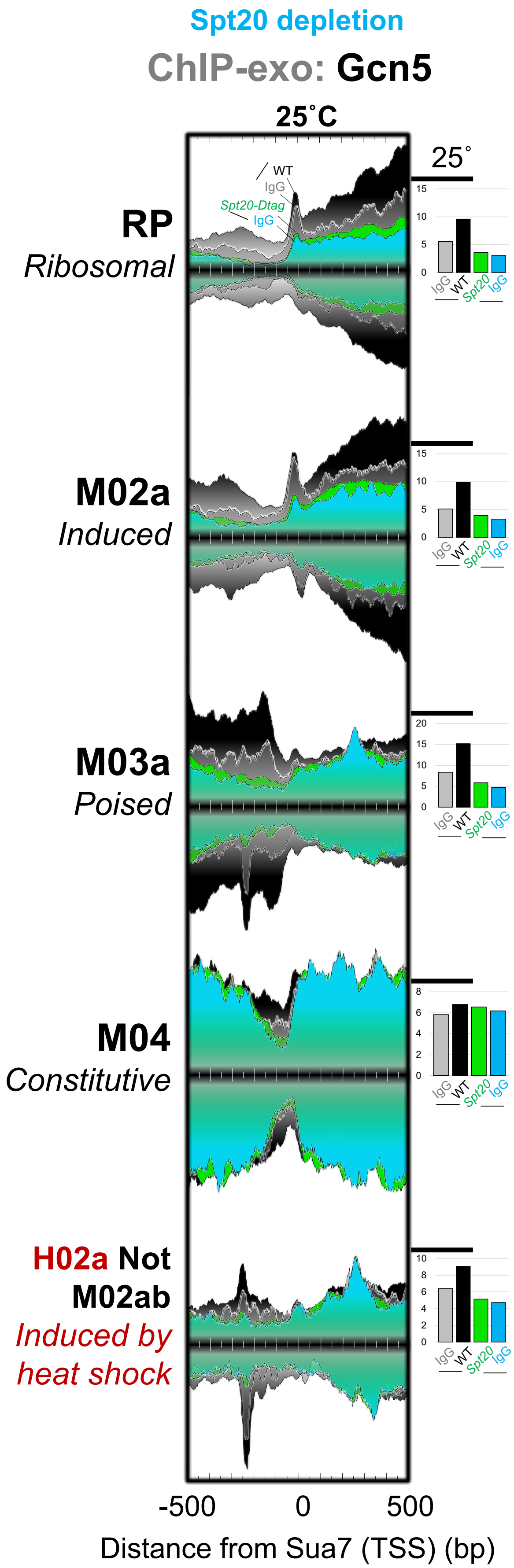

Supplemental Fig. S6. Gcn5 occupancy at inducible promoters depends on SAGA (Spt20). Composite plots of tag 5' ends from ChIP-exo of Gcn5 are distributed around the Sua7 (TSS) reference point within each promoter class. See Fig. 1 and Methods for plotting details. The y-axis occupancy scale is linear and the same within a promoter class for mutant and WT and quantified in the bar graphs to the right. The y-axis occupancy is scaled separately among promoter classes to maximize visual distinction and thus are not directly comparable. Bar graph quantification is relative to the reference point (Sua7) from -500 to -200 on the top (TSS) strand plus from -400 to -100 on the opposite strand (analysis ID: CM31).

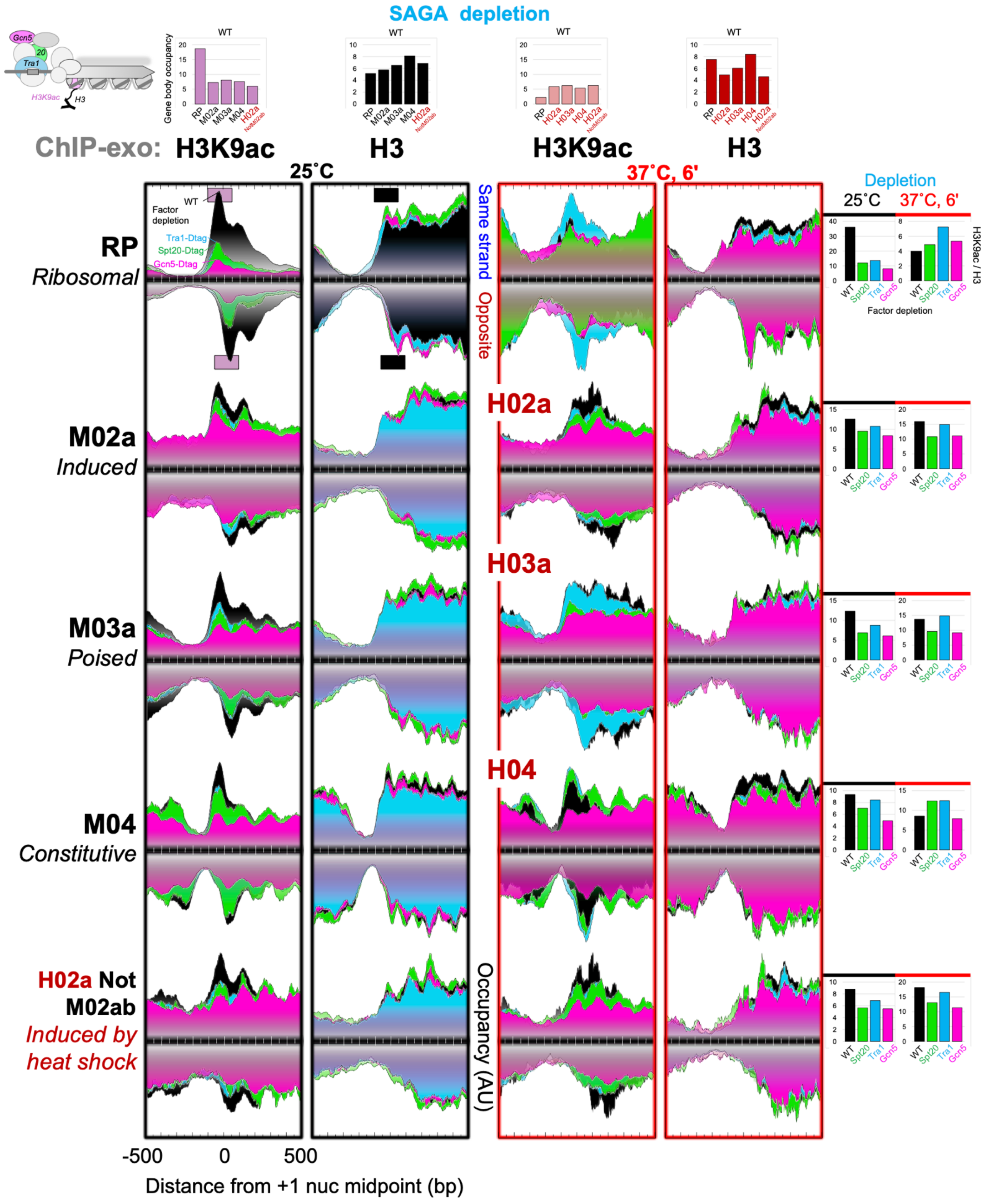

Supplemental Fig. S7. H3K9ac density upon depletion of SAGA subunits. Related to Fig. 5. Composite plots are shown of tag 5' ends from ChIP-exo of H3K9ac and H3 upon depletion of SAGA (Spt20, Tra1, or Gcn5) subunits, distributed around the +1 nucleosome midpoint within each promoter class at the indicated cellular temperature (25 ˚C; 37 ˚C for 6 min.). See Fig. 1 and Methods for plotting details. Quantification bar graphs to the right show H3K9ac/H3 density ratios at +1 nucleosomes assessed from -100 to +50 on the top strand and -50 to +100 on the bottom strand (analysis ID: CM101, CM104, CM107, CM110).
