# Supplementary material for: An integrated SAGA and TFIID PIC assembly pathway selective for poised and induced promoters
Source: Genes Dev. 2022 Sep 1;36(17-18):985–1001. doi: 10.1101/gad.350026.122 (PMC9732905; doi:10.1101/gad.350026.122)
Supplement: Supplemental Material [file supp_gad.350026.122_Supplemental_Material_Content.pdf]

Supplemental Material

Supplemental methods

- *Explanation of reference points*
- *Explanation of promoter class membership criteria and rationale*
- *Explanation of experimental system and controls for measuring SAGA and TFIID dependency*
- *Explanation of discrepancies with Baptista et al. 2017*

Supplemental figures

- *Supplemental_Figure_S1.xlsx – Related to Figure 1*
- *Supplemental_Figure_S2.xlsx – Related to controls*
- *Supplemental_Figure_S3.xlsx – Related to Figure 3*
- *Supplemental_Figure_S4.xlsx – Related to controls*
- *Supplemental_Figure_S5.xlsx – Related to Figure 3*
- *Supplemental_Figure_S6.xlsx – Related to controls*
- *Supplemental_Figure_S7.xlsx – Related to Figure 5*

Supplemental tables

- *Supplemental_Table_S1.xlsx – Genomic feature list*
- *Supplemental_Table_S2.xlsx – Related to Figure 1*
- *Supplemental_Table_S3.xlsx – Related to Figure 1*
- *Supplemental_Table_S4.xlsx – Metadata for genome-wide datasets*
