# Supplementary material for: An integrated SAGA and TFIID PIC assembly pathway selective for poised and induced promoters
Source: Genes Dev. 2022 Sep 1;36(17-18):985–1001. doi: 10.1101/gad.350026.122 (PMC9732905; doi:10.1101/gad.350026.122)
Supplement: Supplemental Material [file supp_gad.350026.122_Supplemental_Methods.pdf]

Supplemental Methods

Chitvan Mittal, Olivia Lang, William K.M. Lai, and B. Franklin Pugh (2022)
An integrated SAGA and TFIID PIC assembly pathway selective for poised and induced promoters

Reference points

TFIIB (Sua7, PEGR ID 12275) locations defined by ChExMix (Yamada et al., 2020) were used as PIC reference points (Rossi et al., 2021; Supplemental Table S1, column G). The Sua7 ChExMix peaks were associated with the closest annotated TSS (Rossi et al. 2021; Xu et al., 2009; Supplemental Table S1, column H) and then the set was filtered to retain the closest Sua7 peak within a -100 to +61 bp window around the TSS. These limits were intended to accommodate variance in annotated PIC and TSS locations while minimizing capture of adjacent PICs. Due to Pol II scanning, a TSS does not always align with a PIC. Therefore, Sua7 peaks were preferred over annotated TSSs. If more than one TSS was associated with the same Sua7 peak, then only the TSS closest to the Sua7 peak was assigned. For all remaining genes without an assigned Sua7 ChExMix peak, the Sua7 annotation defaulted to the TSS annotation. For targets that were enriched at the +1 nucleosome, the +1 nucleosome dyad defined by H3/H4 MNase-seq (Rossi et al. 2021) was used as a reference point (Supplemental Table S1, column I). For targets that bound to the UAS region, the UAS as defined by (Rossi et al., 2021) was used (Supplemental Table S1, column E). In promoter cases where a UAS was not identified, we instead used a distance that was 150 bp upstream (more 5’) of the TSS location (Supplemental Table S1, column D). This distance was based on the average distance that SAGA (Spt7, PEGR ID 11960) ChIP-exo peaks were located upstream of a TSS location.

Promoter class membership criteria and rationale

We started with 5,378 PIC-containing promoters having any of the following labels from (Supplemental Data 1, column D of (Rossi et al., 2021)): 01_RP, 02_STM, 03_TFO, or 04_UNB (Supplemental Table S1, column M). Of these, we removed the 137 ribosomal protein (RP) genes and established them as the RP class. They were labeled based on gene ontologies as encoding a subunit of cytosolic ribosomes (RPL and RPS gene names).

Two sets of induced, poised, and constitutive promoter classes were generated to reflect occupancy data collection under two different environmental conditions: M-series (algorithm IDs: M02a, M03a, and M04) for 25˚C, and H-series (algorithm IDs: H02a, H03a, and H04) for acute heat shock (37˚C, 6 min). This allowed acute reprogramming to be compared to steady-state induction. Their membership was capped at 150 promoters. Capping membership at 150 promoters reflects the approximate number of promoters that were previously defined as having unusually high GTF:TFIID occupancy ratios (Rossi et al., 2021), which is a property of induced promoters (Huisinga and Pugh, 2004; Kuras et al., 2000). This number is consistent with reported numbers of SAGA-regulated genes (Huisinga and Pugh, 2004; Lenstra et al., 2011).

M/H-series algorithm details. Occupancy values for Sua7, Taf2, and Spt7 were calculated for each of 5,378 genes under both heat shock and mock heat shock conditions. Using an offset tag summing strategy, the occupancy counts for Sua7 (PEGR ID: 12275 (25˚C); PEGR ID: 26344 (37˚C, 6 min)) and Taf2 (PEGR ID: 11846 (25˚C); PEGR ID: 28736 (37˚C, 6 min)) for each gene

were the sum of 5' end tags in a 100 bp window on the gene's coding sense strand and antisense strand centered on -26 bp and -6 bp from the center of the Sua7 reference point, respectively. These offsets from the Sua7 reference point reflect where strand-specific signals were at their maximum when examining a composite of all 5,378 promoters. The occupancy counts for Spt7 (PEGR ID: 11960 (25˚C); PEGR ID: 20115 (37˚C, 6 min)) were similarly calculated applying the same offset strategy at the UAS coordinates (or imputed equivalent) instead of the PIC coordinates (Supplemental Table S1, column D).

The induced class (Algorithm IDs: M02 and H02) comprised nonexclusive promoter sets (see Fig. below). Membership in either class required a promoter to 1) not be RP, 2) be among the top 1000 for TFIIB (Sua7) occupancy (i.e., high PIC occupancy) at their respective temperatures, and 3) of these, also being the top 150 for Sua7:Taf2 occupancy ratios at their respective temperatures. M02a and H02a had 62/150 members in common. We also created, but did not extensively analyze, a secondary "buffer" set in each category (Algorithm IDs: M02b and H02b) that represented the penultimate set of 150 promoters when applying M02a or H02a Sua7:Taf2 ratio filters. The intent was to provide a broader separation between M/H02a and M/H03.

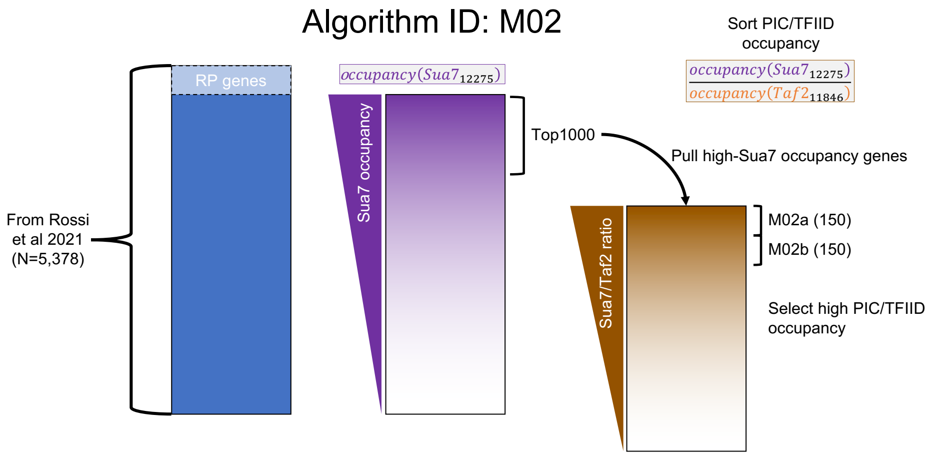

The poised class (Algorithm IDs: M03a and H03a) also comprised two nonexclusive promoter sets. This category was derived from our observation that there were many more promoters having high SAGA occupancy than having high Sua7:Taf2 occupancy ratios (Rossi et al., 2021). This suggested that many promoters may be recruiting SAGA but are not induced. We therefore examined the 150 top-most SAGA-bound promoters at 25˚C (M03a) or upon acute heat shock (H03a) after first removing RP, and the respective M02a,b or H02a,b sets. Consequently, a total of 437 promoters were removed before defining each poised (03a) set. We also created, but did not extensively analyze, a secondary "buffer" set in each category (M03b and H03b) that represented the penultimate set of 150 promoters when applying M03a or H03a SAGA enrichment filters. The intent was to provide a broader separation been M/H03a and M/H04.

Algorithm ID: M03 and M04

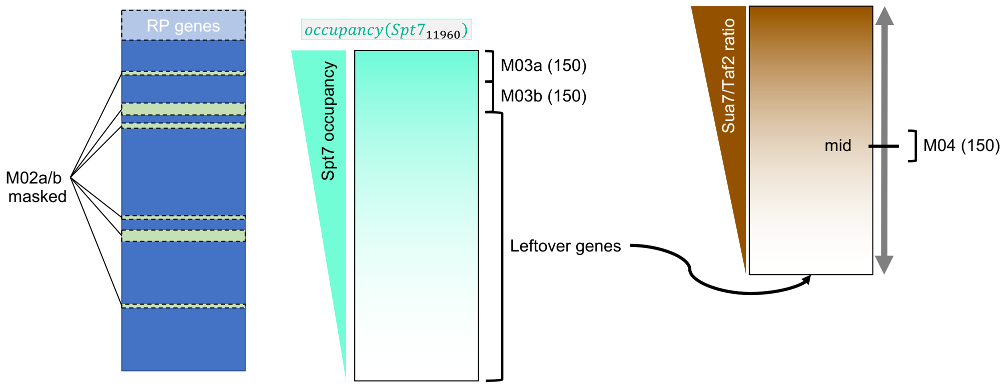

The constitutive group (M04 or H04) comprised two nonexclusive sets of 150 promoters. Membership was defined by first excluding 737 promoters having the following membership: RP, induced (M02a,b or H02a,b respectively), or poised (M03a,b or H03a,b respectively). The constitutive class was further selected to be the 150 promoters being closest to the genome-wide median for Sua7:Taf2 occupancy ratios at their respective temperatures, so as to maximize selection for constitutive character.

The condition-specific heat shock induced class (H02aNotM02ab) comprised a single set of 72 genes. They were not induced at 25˚C (i.e., not M02a,b) but were induced at 37˚C, 6 min (i.e., H02a). The purpose of this set was to evaluate, at the same set of promoters, the involvement of factors prior to and immediately upon induction.

The inducible sets (M/H02,3) are most similar to the following other classifications: STM (Rossi et al., 2021), coactivator-redundant (Donczew et al., 2020), fragile -1 nucleosomes (Kubik et al., 2015), TAF-depleted (Rhee and Pugh, 2012), TATA-containing (Rhee and Pugh, 2012), SAGA-dominated (Huisinga and Pugh, 2004), and TAF-independent (Kuras et al., 2000; (Supplemental Table S1, column M-R). Combining these previous classifications and our new classification, we imputed inducible vs constitutive promoter labels (Supplemental Table S1, column AR). Of the inducible label, we imputed those that were induced vs poised based on Sua7/Taf2 ratios, Sua7 occupancy, and mRNA expression levels (Supplemental Table S1, column AS). We further labeled those that were induced based on high Sua7/Taf2 ratios (M02-like) vs normal ratios (RP-like; Supplemental Table S1, column AT). We emphasize that these labels and class memberships (e.g. M02a, etc.) are a product of algorithmic assignments and thus dependent on the algorithm. As such, promoter assignments will include false positives and false negatives. Thus, for example, the POP1 gene is a member of the M02a induced class but is labeled as constitutive, thereby requiring further investigation.

Experimental system and controls for measuring SAGA and TFIID dependency

Spt20 and Taf1 subunits represent the central core of SAGA/SLIK and TFIID, respectively (Bai et al., 1997; Moqtaderi et al., 1998; Sterner et al., 1999), and so we focused on them. We cannot exclude the possibility that other functionally distinct complexes also contain these subunits, although none are known to exist. Their depletion is expected to impact the entirety of SAGA or TFIID. For example, depletion of Spt20 resulted in the loss of the distinct SAGA module subunit Gcn5 at the UAS of inducible promoters (Supplemental Fig. S6). Translocation

of Spt20 and Taf1 from the nucleus to the cytoplasm occurred upon addition of rapamycin (Supplemental Fig. S2A). Cells grew normally under non-depletion conditions (Supplemental Fig. S2B), indicating that the D-tag had not appreciably affected their function. Indole-3-acetic acid had small effects on growth and rapamycin had no effect on growth of the parental strain (AB001) which lacked the D-tag but had the components necessary for depletion and degradation (Supplemental Fig. S2B).

ChIP-exo exonuclease stop sites for TBP were concentrated and positioned within a few bp of the PIC reference point defined by TFIIB (Sua7; Fig. 1K), thereby validating the PIC position. Positional variance of stop site locations within replicates were <10 bp (Supplemental Fig. S2C). The standard deviation of TBP occupancy amongst replicates in each class was <10% of the mean.

The heat shock response was not substantially affected in the parental AB001 strain or by depletion treatment as evidenced by normal Hsf1 and SAGA binding when compared to BY4741 controls (Supplemental Fig. S4), (Fig. 2A,B). Depletion drugs were added 30 minutes prior to an acute 37˚C heat shock or a 25˚C mock treatment to measure dependency of events on the depleted factors. These drugs alone had little effect on TBP occupancy at 30 minutes, although cells did mount a transient stress response (Supplemental Fig. S3A, 7.5 min). Incubations at those temperatures were maintained for 6 minutes, after which formaldehyde crosslinking was initiated. This experimental design differs from a related one in which heat shock was first performed then followed by SAGA depletion (Warfield et al., 2017). The different order of events in that study do not allow for a direct comparison. Optimal depletion timing was determined in the current study after conducting a depletion time course of Spt20 and separately for Spt7, and compared to the parent strain (Supplemental Fig. S3A; Badjatia et al., 2021).

Discrepancies with Baptista et al., (2017)

Baptista et al., (2017) presented evidence for SAGA being a general cofactor for PIC assembly at most or all yeast promoters. This notion is largely based on the ChEC-seq chromatin binding assay and genome-wide newly synthesized mRNA via 4-thiouracil pulse labeling in SAGA deletion strains (Baptista et al., 2017; Bonnet et al., 2014). However, re-analysis of this data demonstrated that most of these putative genome-wide interactions were not distinct from background control MNase digestion (Mittal et al., 2021).

We also inspected published microarray data on 4-thiouracil pulse-labeling of nascent RNA (Baptista et al., 2017), and found that the negative control Pol I transcription units (rRNA) within the same *spt20Δ* datasets showed at least as much dependence on SAGA as the Pol II transcription units (Supplemental Fig. S5B). Since SAGA is specific to Pol II, specificity of the assay towards SAGA was not demonstrated in these 4-thiouracil experiments. We note that 4-thiouracil assimilation is dependent on FUR1 (uracil phosphoribosyltransferase), whose expression is highly dependent on SAGA (Huisinga and Pugh, 2004). This raises the possibility that SAGA deletion mutants have deficiencies in 4-thiouracil assimilation, which would impact all three RNA polymerase systems. This interpretation is consistent with SAGA deletion strains showing widespread loss of 4-thiouracil incorporation, whereas rapid (30 min) depletion of SAGA has only ~13% of all genes being affected in the same assay (Donczew et al., 2020). This latter observation fits more with long-standing view that only a small subset of yeast genes is

directly SAGA-regulated (Huisinga and Pugh, 2004). Moreover, analysis of the (Baptista et al., 2017) spt20Δ nascent RNA datasets and the (Huisinga and Pugh, 2004) SAGA (spt3Δ) datasets across our five promoter classes, revealed the same trend of SAGA dependency observed in the current study that measures PIC occupancy and nascent or steady-state mRNA production: (Supplemental Fig. S5B, lower panel) compared to (Fig. 3, Supplemental Fig. S3B, and Supplemental Fig. S5C). We further note that strains grow more slowly when deleted or depleted of SAGA (Supplemental Fig. S2B), and thus are expected to have global decreases in PIC and Pol I, II, and III assembly that may be more of a function of growth rates (i.e., indirect effects) than global defects in SAGA function.

Supplemental References

Badjatia, N., Rossi, M.J., Bataille, A.R., Mittal, C., Lai, W.K.M., and Pugh, B.F. (2021). Acute stress drives global repression through two independent RNA polymerase II stalling events in Saccharomyces. Cell Rep 34, 108640.

Bai, Y., Perez, G.M., Beechem, J.M., and Weil, P.A. (1997). Structure-function analysis of TAF130: identification and characterization of a high-affinity TATA-binding protein interaction domain in the N terminus of yeast TAF(II)130. Mol Cell Biol 17, 3081-3093.

Baptista, T., Grunberg, S., Minoungou, N., Koster, M.J.E., Timmers, H.T.M., Hahn, S., Devys, D., and Tora, L. (2017). SAGA Is a General Cofactor for RNA Polymerase II Transcription. Mol Cell 68, 130-143 e135.

Bonnet, J., Wang, C.Y., Baptista, T., Vincent, S.D., Hsiao, W.C., Stierle, M., Kao, C.F., Tora, L., and Devys, D. (2014). The SAGA coactivator complex acts on the whole transcribed genome and is required for RNA polymerase II transcription. Genes Dev 28, 1999-2012.

Donczew, R., Warfield, L., Pacheco, D., Erijman, A., and Hahn, S. (2020). Two roles for the yeast transcription coactivator SAGA and a set of genes redundantly regulated by TFIID and SAGA. Elife 9.

Huisinga, K.L., and Pugh, B.F. (2004). A genome-wide housekeeping role for TFIID and a highly regulated stress-related role for SAGA in Saccharomyces cerevisiae. Mol Cell 13, 573-585.

Kubik, S., Bruzzone, M.J., Jacquet, P., Falcone, J.L., Rougemont, J., and Shore, D. (2015). Nucleosome Stability Distinguishes Two Different Promoter Types at All Protein-Coding Genes in Yeast. Mol Cell 60, 422-434.

Kuras, L., Kosa, P., Mencia, M., and Struhl, K. (2000). TAF-Containing and TAF-independent forms of transcriptionally active TBP in vivo. Science 288, 1244-1248.

Lenstra, T.L., Benschop, J.J., Kim, T., Schulze, J.M., Brabers, N.A., Margaritis, T., van de Pasch, L.A., van Heesch, S.A., Brok, M.O., Groot Koerkamp, M.J., et al. (2011). The specificity and topology of chromatin interaction pathways in yeast. Mol Cell 42, 536-549.

Mittal, C., Rossi, M.J., and Pugh, B.F. (2021). High similarity among ChEC-seq datasets. bioRxiv https://doi.org/10.1101/2021.02.04.429774.

Moqtaderi, Z., Keaveney, M., and Struhl, K. (1998). The histone H3-like TAF is broadly required for transcription in yeast. Mol Cell 2, 675-682.

Rhee, H.S., and Pugh, B.F. (2012). Genome-wide structure and organization of eukaryotic pre-initiation complexes. Nature 483, 295-301.

Rossi, M.J., Kuntala, P.K., Lai, W.K.M., Yamada, N., Badjatia, N., Mittal, C., Kuzu, G., Bocklund, K., Farrell, N.P., Blanda, T.R., et al. (2021). A high-resolution protein architecture of the budding yeast genome. Nature 592, 309-314.

Sterner, D.E., Grant, P.A., Roberts, S.M., Duggan, L.J., Belotserkovskaya, R., Pacella, L.A., Winston, F., Workman, J.L., and Berger, S.L. (1999). Functional organization of the yeast SAGA complex: distinct components involved in structural integrity, nucleosome acetylation, and TATA-binding protein interaction. Mol Cell Biol 19, 86-98.

Warfield, L., Ramachandran, S., Baptista, T., Devys, D., Tora, L., and Hahn, S. (2017). Transcription of Nearly All Yeast RNA Polymerase II-Transcribed Genes Is Dependent on Transcription Factor TFIID. Mol Cell 68, 118-129 e115.

Xu, Z., Wei, W., Gagneur, J., Perocchi, F., Clauder-Munster, S., Camblong, J., Guffanti, E., Stutz, F., Huber, W., and Steinmetz, L.M. (2009). Bidirectional promoters generate pervasive transcription in yeast. Nature 457, 1033-1037.

Yamada, N., Kuntala, P.K., Pugh, B.F., and Mahony, S. (2020). ChExMix: A Method for Identifying and Classifying Protein-DNA Interaction Subtypes. J Comput Biol 27, 429-435.
